# An Auxiliary Variable Method for Markov Chain Monte Carlo Algorithms in High Dimension

**DOI:** 10.3390/e20020110

**Published:** 2018-02-07

**Authors:** Yosra Marnissi, Emilie Chouzenoux, Amel Benazza-Benyahia, Jean-Christophe Pesquet

**Affiliations:** 1 SSAFRAN TECH, Groupe Safran, 78772 Magny-les-Hameaux, France; 2 Laboratoire Informatique Gaspard Monge (LIGM)-UMR 8049 CNRS, University Paris-East, 93162 Noisy-le-Grand, France; 3 Center for Visual Computing, University Paris-Saclay, 91190 Gif-sur-Yvette, France; 4 COSIM Research Laboratory, Higher School of Communication of Tunis (SUP’COM), University of Carthage, 2083 Ariana, Tunisia

**Keywords:** data augmentation, auxiliary variables, MCMC, Gaussian models, large scale problems, Bayesian methods

## Abstract

In this paper, we are interested in Bayesian inverse problems where either the data fidelity term or the prior distribution is Gaussian or driven from a hierarchical Gaussian model. Generally, Markov chain Monte Carlo (MCMC) algorithms allow us to generate sets of samples that are employed to infer some relevant parameters of the underlying distributions. However, when the parameter space is high-dimensional, the performance of stochastic sampling algorithms is very sensitive to existing dependencies between parameters. In particular, this problem arises when one aims to sample from a high-dimensional Gaussian distribution whose covariance matrix does not present a simple structure. Another challenge is the design of Metropolis–Hastings proposals that make use of information about the local geometry of the target density in order to speed up the convergence and improve mixing properties in the parameter space, while not being too computationally expensive. These two contexts are mainly related to the presence of two heterogeneous sources of dependencies stemming either from the prior or the likelihood in the sense that the related covariance matrices cannot be diagonalized in the same basis. In this work, we address these two issues. Our contribution consists of adding auxiliary variables to the model in order to dissociate the two sources of dependencies. In the new augmented space, only one source of correlation remains directly related to the target parameters, the other sources of correlations being captured by the auxiliary variables. Experiments are conducted on two practical image restoration problems—namely the recovery of multichannel blurred images embedded in Gaussian noise and the recovery of signal corrupted by a mixed Gaussian noise. Experimental results indicate that adding the proposed auxiliary variables makes the sampling problem simpler since the new conditional distribution no longer contains highly heterogeneous correlations. Thus, the computational cost of each iteration of the Gibbs sampler is significantly reduced while ensuring good mixing properties.

## 1. Introduction

In a wide range of applicative areas, we do not have access to the signal of interest $\bar{x}\in R^{Q}$, but only to some observations $z\in R^{N}$ related to $\bar{x}$ through the following model:

(1) $$z=D(H\bar{x}),$$

where $H\in R^{N\times Q}$ is the observation matrix that may express a blur or a projection and D is the noise model representing measurement errors. In this paper, we are interested in finding an estimator $\hat{x}$ of $\bar{x}$ from the observations z. This inverse problem arises in several signal processing applications, such as denoising, deblurring, and tomography reconstruction [1,2].

The common Bayesian procedure for signal estimation consists of deriving estimators from the posterior distribution that captures all information inferred about the target signal from the collected data. Given the observation model (1), the minus logarithm of the density posterior distribution reads:

(2) $$\left(\forall x\in R^{Q}\right)\;J\left(x\right)=-\log p\left(x|z\right)=\Phi \left(Hx;z\right)+\Psi \left(Vx\right).$$

Hereabove, Φ is the neg-log likelihood that may take various forms depending on the noise statistical model D. In particular, if D models an additive Gaussian noise with covariance $\Lambda^{-1}$, it reduces (up to an additive constant) to the least squares function $\Phi \left(Hx;z\right)=\frac{1}{2}{∥Hx-z∥}_{\Lambda }^{2}$. Other common choices can be found for instance in [3,4]. Moreover, Ψ(V·) is related to some prior knowledge one can have about x, and $V\in R^{M\times N}$ is a linear transform that can describe, for example, a frame analysis [5] or a discrete gradient operator [6]. Within a Bayesian framework, it is related to a prior distribution of density p(x) whose logarithm is given by logp(x)=−Ψ(Vx).

Monte Carlo inference approaches allow us to have a good description of the target space from a set of samples drawn from a distribution [7,8,9,10,11,12]. In particular, these samples can be used to infer useful statistics such as the mean and the variance. In the context of Bayesian estimation, these techniques appear useful to compute, for example, the minimum mean square error (MMSE) estimator, which is equivalent to the posterior mean. In this case, the MMSE estimator is approximated using the empirical average over the generated samples from the posterior distribution. When the exact expression of the posterior density is intractable, Markov chain Monte Carlo (MCMC) algorithms have been widely used to approximate it [13]. These techniques are random variable generators that allow us to draw samples from complicated distributions. Perhaps the most commonly used MCMC algorithm is the Metropolis–Hastings (MH), which operates as follows [14]: from a given proposal distribution, we construct an irreducible Markov chain whose stationary distribution is the sought posterior law (i.e., samples generated by the algorithm after a suitable burn-in period are distributed according to desired posterior law). At each iteration *t*, a decision rule is applied to accept or reject the proposed sample given by the following acceptance probability:

(3) $$\alpha \left(x^{\left(t\right)},{\tilde{x}}^{\left(t\right)}\right)=\min\left(1,\frac{p\left({\tilde{x}}^{\left(t\right)}|z\right)g\left(x^{\left(t\right)}|{\tilde{x}}^{\left(t\right)}\right)}{p\left(x^{\left(t\right)}|z\right)g\left({\tilde{x}}^{\left(t\right)}|x^{\left(t\right)}\right)}\right),$$

where ${\tilde{x}}^{\left(t\right)}$ is the proposed sample at iteration *t*, generated from a proposal distribution with density $g(.|x^{\left(t\right)})$ that may depend on the current state $x^{\left(t\right)}$. Note that when more than one unknown variable needs to be estimated (e.g., acquisition parameters or prior hyperparameters), one can iteratively draw samples from the conditional posterior distribution for each variable given the remaining ones using an MH iteration. This is known as the hybrid Gibbs sampler [15]. High-dimensional models—often encountered in inverse problems (e.g., in multispectral remote sensing applications [16])—constitute a challenging task for Bayesian inference problems. While many popular sampling algorithms have been widely used to fit complex multivariable models in small-dimensional spaces [17,18,19,20,21,22], they generally fail to explore the target distribution efficiently when applied to large-scale problems, especially when the variables are highly correlated. This may be due to the poor mixing properties of the Markov chain or to the high computational cost of each iteration [17].

In this work, we propose a novel approach based on a data augmentation strategy [23] which aims at overcoming the limitations of standard Bayesian sampling algorithms when facing large-scale problems. The remainder of this paper is organized as follows. In Section 2, we discuss the main difficulties encountered in standard sampling methods for large-scale problems. We show how the addition of auxiliary variables to the model can improve their robustness with respect to these issues. The core of our contribution is detailed in Section 3. We first give a complete description of the proposed approach in the case of Gaussian noise, and we study its extension to scale mixtures of Gaussian models. Furthermore, we demonstrate how the proposed approach can facilitate sampling from Gaussian distributions in Gibbs algorithms. Then, some computational issues arising in the proposed Bayesian approach are discussed. Section 4 and Section 5 are devoted to the experimental validation of our method. In Section 4, we show the advantages of the proposed approach in dealing with high-dimensional models involving highly correlated variables over a dataset of multispectral images affected by blur and additive Gaussian noise. In Section 5, we test the performance of our method in sampling from large-scale Gaussian distributions through an application to image recovery under two-term mixed Gaussian noise. Finally, we give some conclusions and perspectives in Section 6.

## 2. Motivation

### 2.1. Sampling Issues in High-Dimensional Space

MCMC sampling methods may face two main difficulties when applied to large-scale inverse problems. First, except for particular cases (e.g., circulant observation matrix), the structure of the observation model that links the unknown signal to the observations usually makes the estimation of the parameters of the posterior distribution quite involved. Second, even with simple models, the posterior distribution may still be difficult to sample from directly or to explore efficiently using standard sampling algorithms. As a specific case, this problem arises for Gaussian distributions if the problem dimension is too high [24]. It can also arise in MH algorithms when sophisticated proposal rules are employed with the aim of coping with both the high dimensionality and the strong correlation existing between the target parameters [22]. In what follows, we will give more details about these two contexts.

#### 2.1.1. Sampling from High-Dimensional Gaussian Distribution

Let us focus on the problem of sampling from a multivariate Gaussian distribution with a given precision matrix $G\in R^{Q\times Q}$. This problem emerges in many applications, such as linear inverse problems involving Gaussian or hierarchical Gaussian models. More precisely, let us consider the following linear model:

(4) z=Hx+w,

where w is $R^{N}$-valued, and let us assume that conditionally to some latent variables, w and x are drawn from Gaussian distributions $N(0_{N},\Lambda^{-1})$, and $N(m_{x},G_{x}^{-1})$, respectively, where $m_{x}\in R^{Q}$, $\Lambda \in R^{N\times N}$, and $G_{x}\in R^{Q\times Q}$ are positive semi-definite matrices. In the following, when not mentioned, the Gaussian law can be degenerated; that is, the precision matrix is semi-definite positive but not with full rank. In this case, ${\left(\cdot \right)}^{-1}$ denotes the generalized inverse. The parameters of these Gaussian distributions may be either fixed or unknown (i.e., involving some unknown hyperparameters such as regularization or acquisition parameters). It follows that the posterior distribution of x is Gaussian, with mean $m\in R^{Q}$ and precision matrix $G\in R^{N\times N}$ defined as follows:

(5) $$G=H^{⊤}\Lambda H+G_{x}$$

(6) $$m=G^{-1}\left(H^{⊤}\Lambda z+G_{x}m_{x}\right).$$

A common solution to sample from $N(m,G^{-1})$ is to use the Cholesky factorization of the covariance or the precision matrix G [25]. However, when implemented through a Gibbs sampler, this method is of limited interest. First, the precision matrix G may depend on the unknown parameters of the model and may thus take different values along the algorithm. Thereby, spending such high computational time at each iteration of the Gibbs sampler to compute the Cholesky decomposition of the updated matrix may be detrimental to the convergence speed of the Gibbs sampler. Another concern is that when dealing with high dimensional problems, we generally have to face not only computational complexity issues but also memory limitations. Such problems can be alleviated when the matrix presents some specific structures (e.g., circulant [26,27] or sparse [28]). However, for more complicated structures, the problem remains critical, especially when $H^{⊤}\Lambda H$ and $G_{x}$ cannot be diagonalized in the same basis. Other recently proposed algorithms for sampling Gaussian distributions in high dimension follow a two-step perturbation-optimization approach [24,29,30,31,32,33], which can be summarized as follows:

- Perturbation: Draw a Gaussian random vector $n_{1}∼N\left(0_{Q},G\right)$.
- Optimization: Solve the linear system $Gn_{2}=n_{1}+H^{⊤}\Lambda z+G_{x}m_{x}$.

The solution to the above linear system can be approximated using iterative methods such as conjugate gradient algorithms, leading to an approximate sample of the sought distribution [30,31]. This issue has been considered in [32] by adding a Metropolis step in the sampling algorithm. In [24,33], the authors propose to reduce the computational cost by sampling along mutually conjugate directions instead of the initial high-dimensional space.

#### 2.1.2. Designing Efficient Proposals in MH Algorithms

Non-Gaussian models arise in numerous applications in inverse problems [34,35,36,37]. In this context, the posterior distribution is non-Gaussian and does not generally follow a standard probability model. In this respect, MH algorithms are good tools for exploring such posteriors, and hence for drawing inferences about models and parameters. However, the challenge for MH algorithms is constructing a proposal density that provides a good approximation of the target density while being inexpensive to manipulate. Typically, in large-scale problems, the proposal distribution takes the form of a random walk (RW); that is, in each iteration, the proposal density $g(.|x^{\left(t\right)})$ in (3) is a Gaussian law centered at the current state $x^{\left(t\right)}$ and with covariance matrix $\varepsilon^{2}Q\left(x^{\left(t\right)}\right)$. Moreover, ε is a positive constant whose value is adjusted so that the acceptance probability in (3) is bounded away from zero at convergence [17]. Other sampling algorithms incorporate information about the derivative of the logarithm of the target distribution to guide the Markov chain toward the target space where samples should be mostly concentrated. For instance, when the target density is differentiable, one can use Langevin-based algorithms where the mean of the Gaussian proposal density is replaced with one iteration of a preconditioned gradient descent algorithm as follows [20,22,38,39,40,41]:

(7) $${\tilde{x}}^{\left(t\right)}∼N\left(x^{\left(t\right)}-\frac{\varepsilon^{2}}{2}Q{\left(x^{\left(t\right)}\right)}^{-1}\nabla J\left(x^{\left(t\right)}\right),\varepsilon^{2}Q{\left(x^{\left(t\right)}\right)}^{-1}\right).$$

In (7), ∇J is the gradient of J, ε is a positive constant, and Q is a symmetric definite positive matrix that captures possible correlations between the coefficients of the signal. Note that some advanced versions of Langevin-based algorithms have been proposed to address problems with non-smooth laws [42,43]. It is worth noting that the choice of the scale matrices ${\left(Q(x^{\left(t\right)})\right)}_{t}$ may deeply affect the efficiency of the aforementioned algorithms [22]. In fact, an inappropriate choice of Q may alter the quality of the Markov chain, leading to very correlated samples and thereby biased estimates. Moreover, computationally cheap matrices are also preferable, especially in high-dimensional spaces. In the case of low-dimensional problems and when the coefficients of the signal are not highly correlated, the standard RW and Metropolis-adapted Langevin algorithm (MALA) obtained for $Q\equiv I_{Q}$ achieve overall good results. For instance, in the context of denoising problems with uncorrelated Gaussian noise, when the coefficients of the signal are assumed to be statistically independent in the prior, they can either be sampled independently using RW or jointly by resorting to MALA. However, these algorithms may be inaccurate for large-scale problems, especially when the coefficients of the signal exhibit high correlations [22]. In this case, the design of a good proposal often requires consideration of the curvature of the target distribution. More sophisticated (and thus more computationally expensive) scale matrices should be chosen to drive the chain in the directions that reflect the dependence structure. Optimally, the curvature matrix should be chosen such that it adequately captures two kinds of dependencies: correlation over the observations specified by the observation model, and correlation between different coefficients of the target signal specified by the prior law. For instance, Q can be set to the Hessian matrix of the minus logarithm of the posterior density in the current state [20,21], or to the Fisher matrix (especially when the Hessian matrix is not definite positive [22,41]) or to the empirical covariance matrix computed according to the previous states of the Markov chain [44]. When the minus-log of the target density can be expressed as in (2), good candidates of the curvature matrix take the following form:

(8) $$Q=H^{⊤}\Lambda H+V^{⊤}\Omega V\;,$$

where Λ and Ω are semi-definite positive matrices. Feasible numerical factorization of Q can be ensured if $H^{⊤}\Lambda H$ and $V^{⊤}\Omega V$ are diagonalizable in the same basis. Otherwise, the use of the full matrix (8) in the scheme (7) remains generally of limited interest, especially for large-scale problems where the manipulation of the resulting proposal generally induces a high computational complexity altering the convergence speed. Alternatively, under mild conditions on the posterior density, the Majorize–Minimize strategy offers a high flexibility for building curvature matrices with a lower computational cost (e.g., diagonal matrices, bloc-diagonal matrices, circulant, etc.) [40]. However, it should be pointed out that MH algorithms with too-simple preconditioning matrices resulting from rough approximations of the posterior density may fail to explore the target space efficiently. Therefore, the scale matrix Q should be adjusted to achieve a good tradeoff between the computational complexity induced in the algorithm and the accuracy/closeness of the proposal to the true distribution.

### 2.2. Auxiliary Variables and Data Augmentation Strategies

It is clear that the main difficulty arising in the aforementioned sampling problems is due to the intricate form of the target covariance matrix making difficult the direct sampling or the construction of a good MH proposal that mimics the local geometry of the target law. More specifically, there are generally heterogeneous types of dependencies between the coefficients of the signal, coming either from the likelihood or from the prior information. For instance, the observation matrix H in the likelihood may bring high dependencies between distant coefficients, even if the latter are assumed to be statistically dependent in the prior law. One solution is to address the problem in another domain where H can be easily diagonalized (i.e., the coefficients of the signal become uncorrelated in the likelihood). However, if one also considers the prior dependencies, this strategy may become inefficient, especially when the prior covariance matrix cannot be diagonalized in the same basis as H, which is the case in most real problems. One should therefore process these two sources of correlations separately.

To improve the mixing of sampling algorithms, many works have proposed the elimination of one of these sources of correlation directly related to x by adding some auxiliary variables to the initial model, associated with a given conditional distribution such that simulation can be performed in a simpler way in the new larger space. Instead of simulating directly from the initial distribution, a Markov chain is constructed by alternately drawing samples from the conditional distribution of each variable, which reduces to a Gibbs sampler in the new space. This technique has been used in two different statistical literatures: data augmentation [45] and auxiliary variables strategies [46]. It is worth noting that the two methods are equivalent in their general formulation, and the main difference is often related to the statistical interpretation of the auxiliary variable (unobserved data or latent variable) [23]. In the following, we will use the term data augmentation (DA) to refer to any method that constructs sampling algorithms by introducing auxiliary variables. Some DA algorithms have been proposed in [47,48,49,50,51,52,53]. Particular attention has been focused on the Hamiltonian MCMC (HMC) approach [22,54], which defines auxiliary variables based on physically-inspired dynamics.

In the following, we propose to alleviate the problem of heterogeneous dependencies by resorting to a DA strategy. More specifically, we propose to add some auxiliary variables $u\in R^{J}$ with predefined conditional distribution of density p(u|x,z)=p(u|x) so that the minus logarithm of the joint distribution density p(x,u|z) can be written as follows:

(9) J(x,u)=J(u|x)+J(x),

where J(u|x)=−logp(u|x) up to an additive constant. Two conditions should be satisfied by p(x,u|z) for the DA strategy to be valid:

$$\begin{array}{cc} \left(C_{1}\right) & \int_{R^{J}}p\left(x,u|z\right)\;du=p(x|z),\\ \left(C_{2}\right) & \int_{R^{Q}}p\left(x,u|z\right)\;dx=p(u|z), \end{array}$$

where p(u|z) should define a valid probability density function (i.e., nonnegative and with integral with respect to u equal to 1). In fact, the importance of Condition $\left(C_{1}\right)$ is obvious, because the latent variable is only introduced for computational purposes and should not alter the considered initial model. The need for the second requirement $\left(C_{2}\right)$ stems from the fact that p(x,u|z) should define the density of a proper distribution. Note that

- the first condition is satisfied thanks to the definition of the joint distribution in (9), provided that p(u|x,z) is a density of a proper distribution;
- for the second condition, it can be noticed that if the first condition is met, Fubini–Tonelli’s theorem allows us to claim that
  (10) $$\int_{R^{J}}\left(\int_{R^{Q}}p\left(x,u|z\right)\;dx\right)\;du=\int_{R^{Q}}\left(\int_{R^{J}}p\left(x,u|z\right)\;du\right)\;dx=\int_{R^{Q}}p\left(x|z\right)\;dx=1.$$
  This shows that p(u|z) as defined in $\left(C_{2}\right)$ is a valid probability density function.

Instead of simulating directly from $P_{x|z}$, we now alternatively draw (in an arbitrary order) samples from the conditional distributions of the two variables x and u of respective densities $P_{x|u,z}$ and $P_{u|x,z}$. This simply reduces to a special case of a hybrid Gibbs sampler algorithm with two variables, where each iteration *t* is composed of two sampling steps which can be expressed as follows:

- Sample $u^{\left(t+1\right)}$ from $P_{u|x^{\left(t\right)},z}$;
- Sample $x^{\left(t+1\right)}$ from $P_{x|u^{\left(t+1\right)},z}$.

Under mild technical assumptions [9,55], the constructed chain ${\left(x^{\left(t\right)},u^{\left(t\right)}\right)}_{t⩾0}$ can be proved to have a stationary distribution $P_{x,u|z}$. The usefulness of the DA strategy is mainly related to the fact that with an appropriate choice of p(u|x,z), drawing samples from the new conditional distributions $P_{x|u,z}$ and $P_{u|x,z}$ is much easier than sampling directly from the initial distribution $P_{x|z}$. Let us emphasize that, for the sake of efficiency, the manipulation of p(u|x,z) must not induce a high computation cost in the algorithm. In this work, we propose the addition of auxiliary variables u to the model such that the dependencies resulting from the likelihood and the prior are separated; that is, J(u|x) is chosen in such a way that only one source of correlations remains related directly to x in p(x,u|z), the other sources of correlations only intervening through the auxiliary variables u and z. Note that the advantage of introducing auxiliary variables in optimization or sampling algorithms has also been illustrated in several works in the image processing literature, related to half quadratic approaches [26,56,57,58,59,60]. This technique has also been considered in [61] in order to simplify the sampling task by using a basic MH algorithm in a maximum likelihood estimation problem. Finally, in [62], a half-quadratic formulation was used to replace the prior distribution, leading to a new posterior distribution from which inference results are deduced.

The contribution of our work is the proposal of an extended formulation of the data augmentation method that was introduced in [60] in the context of variational image restoration under uncorrelated Gaussian noise. Our proposal leads to a novel acceleration strategy for sampling algorithms in large-scale problems.

## 3. Proposed Approach

In this section, we discuss various scenarios typically arising in inverse problems and we explain how our approach applies in these contexts.

### 3.1. Correlated Gaussian Noise

Let us consider the linear observation model (4) when the noise term w is assumed to be Gaussian, additive, and independent from the signal that is $w∼N(0_{N},\Lambda^{-1})$, with $\Lambda \in R^{N\times N}$ a symmetric semi-definite positive precision matrix that is assumed to be known. In this context, the minus logarithm of the posterior density takes the following form:

(11) $$\left(\forall x\in R^{Q}\right)\;J\left(x\right)=\frac{1}{2}{\left(Hx-z\right)}^{⊤}\Lambda \left(Hx-z\right)+\Psi \left(Vx\right).$$

Simulating directly from this distribution is generally not possible, and standard MCMC methods may fail to explore it efficiently due to the dependencies between signal coefficients [22]. In particular, the coupling induced by the matrix $H^{⊤}\Lambda H$ may hinder the construction of suitable proposals when using MH algorithms. For example, when $V=I_{Q}$ and $\Psi \left(x\right)=\sum_{i=1}^{Q}\psi_{i}\left(x_{i}\right)$, RW and standard MALA algorithms may behave poorly, as they do not account for data fidelity dependencies, while a preconditioned MALA approach with full curvature matrices may exhibit high computational load due to the presence of heterogeneous dependencies [39].

In the following, we propose the elimination of the coupling induced by the linear operators (H,Λ) by adding auxiliary variables. Since the data fidelity term is Gaussian, a natural choice is to define p(u|x,z) as a Gaussian distribution with mean Ax and covariance matrix C:

(12) $$p\left(u|x,z\right)=\frac{det{\left(C\right)}^{-1/2}}{{\left(2\pi \right)}^{J/2}}\exp\left(-\frac{1}{2}{∥C^{-1/2}\left(u-Ax\right)∥}^{2}\right)\;,$$

where $C\in R^{J\times J}$ is a symmetric positive definite covariance matrix and $A\in R^{J\times Q}$. Then, the joint distribution satisfies the two conditions $\left(C_{1}\right)$ and $\left(C_{2}\right)$ defined in Section 2, and its minus logarithm has the following expression:

(13) $$\left(\forall x\in R^{Q}\right)\left(\forall u\in R^{J}\right)\;J\left(x,u\right)=\frac{1}{2}\left(x^{⊤}Yx+z^{⊤}\Lambda z+u^{⊤}C^{-1}u-2x^{⊤}\left(H^{⊤}\Lambda z+A^{⊤}C^{-1}u\right)\right)+\Psi \left(Vx\right),$$

with

(14) $$Y=H^{⊤}\Lambda H+A^{⊤}C^{-1}A.$$

The expression in (12) yields the sampling scheme:

(15) $$\left(\forall t\in N\right)\;u^{\left(t+1\right)}=Ax^{\left(t\right)}+C^{1/2}n^{\left(t\right)},$$

with $n^{\left(t\right)}∼N\left(0_{J},I_{J}\right)$. The efficiency of the DA strategy is thus highly related to the choice of the matrices A and C. Under the requirement that C is positive definite, the choice of (A,C) is subjective and is related to specifying the source of heterogeneous dependencies that one wants to eliminate in the target distribution based on the properties of H, Λ, V, and Ψ. More specifically, one should identify if the main difficulty stems from the structure of matrix $H^{⊤}\Lambda H$ or only from the non-trivial form of the precision matrix Λ. In what follows, we will elaborate different solutions according to the type of encountered difficulty.

**Alternative I:** Eliminate the Coupling Induced by Λ

Let us first consider the problem of eliminating the coupling induced by matrix Λ. This problem is encountered for example for Model (5) with circulant matrices H and $G_{x}$ and with $\Lambda \neq I_{N}$, which induces further correlation when passing to the Fourier domain. In this context, we propose the elimination of the correlations induced by Λ by setting

(16) $$Y=\frac{1}{\mu }H^{⊤}H\;,$$

where μ>0 is such that ${\mu ∥\Lambda ∥}_{S}<1$, where ${∥\cdot ∥}_{S}$ denotes the spectral norm. This is equivalent to choosing A and C such that

(17) $$A^{⊤}C^{-1}A=H^{⊤}\left(\frac{1}{\mu }I_{N}-\Lambda \right)H.$$

Note that the condition over μ allows toguarantees that C is positive definite. Under (16), the minus logarithm of the conditional distribution of x given z and u reads, up to an additive constant:

(18) $$\left(\forall x\in R^{Q}\right)\left(\forall u\in R^{J}\right)\;J\left(x|u\right)=\frac{1}{2\mu }{∥Hx∥}^{2}-x^{⊤}\left(H^{⊤}\Lambda z+A^{⊤}C^{-1}u\right)+\Psi \left(Vx\right).$$

Let us discuss the application of the hybrid Gibbs sampling algorithm from Section 2 to this particular decomposition. The sampling scheme (15) yields:

(19) $$\left(\forall t\in N\right)\;A^{⊤}C^{-1}u^{\left(t+1\right)}=A^{⊤}C^{-1}Ax^{\left(t\right)}+A^{⊤}C^{-1/2}n^{\left(t\right)}\;,$$

where $n^{\left(t\right)}∼N\left(0_{J},I_{J}\right)$. Since A and C satisfy (17), this leads to:

(20) $$\left(\forall t\in N\right)\;A^{⊤}C^{-1}u^{\left(t+1\right)}=H^{⊤}\left(\frac{1}{\mu }I_{N}-\Lambda \right)Hx^{\left(t\right)}+A^{⊤}C^{-1/2}n^{\left(t\right)}.$$

We can remark that for every t∈N, $A^{⊤}C^{-1/2}n^{\left(t\right)}$ follows the centered Gaussian distribution with covariance matrix $H^{⊤}\left(\frac{1}{\mu }I_{N}-\Lambda \right)H$. It follows that

(21) $$\left(\forall t\in N^{*}\right)\;A^{⊤}C^{-1}u^{\left(t\right)}=H^{⊤}v^{\left(t\right)}\;,$$

where

(22) $$\left(\forall t\in N\right)\;v^{\left(t+1\right)}∼N\left(\Gamma Hx^{\left(t\right)},\Gamma \right),$$

and $\Gamma =\frac{1}{\mu }I_{N}-\Lambda$ is definite positive by construction. Then, the resulting algorithm can be viewed as a hybrid Gibbs sampler, associated to the minus logarithm of the conditional distribution of x given z and a new auxiliary variable $v∼N\left(\Gamma Hx,\Gamma \right)$:

(23) $$\left(\forall x\in R^{Q}\right)\;J\left(x|v\right)=\frac{1}{2\mu }{∥Hx-\mu \left(\Lambda z+v\right)∥}^{2}+\Psi \left(Vx\right).$$

The main steps of the proposed Gibbs sampling algorithm are given in Algorithm 1. The appealing advantage of this algorithm with respect to a Gibbs sampler which would be applied directly to Model (5) when H and $G_{x}$ are diagonalizable in the same domain is that it allows easy handling of the case when Λ is not equal to a diagonal matrix having identical diagonal elements.

**Algorithm 1** Gibbs sampler with auxiliary variables in order to eliminate the coupling induced by Λ.**Initialize:**
$x^{\left(0\right)}\in R^{Q}$, $v^{\left(0\right)}\in R^{N}$, μ>0 such that ${\mu ∥\Lambda ∥}_{S}<1$1:**for** t=0,1,… **do**2: Generate $v^{\left(t+1\right)}∼N\left(\Gamma Hx^{\left(t\right)},\Gamma \right)$ where $\Gamma =\frac{1}{\mu }I_{N}-\Lambda$3: Generate $x^{\left(t+1\right)}∼P_{x|v^{\left(t+1\right)},z}$4:**end for**


Note that minimizing (23) can be seen as a restoration problem with an uncorrelated noise of variance μ. It can be expected that Step 3 in Algorithm 1 can be more easily implemented in the transform domain where H and V are diagonalized, when this is possible (see Section 5 for an example)

**Alternative II:** Eliminate the Coupling Induced by $H^{⊤}\Lambda H$

In a large class of regularized models, H and V have different properties. While H almost reflects a blur, a projection, or a decimation matrix, V may model a wavelet transform or a discrete gradient operator. Such difference in their properties induces a complicated structure of the posterior covariance matrix. To address such cases, we propose the elimination of the source of correlations related to x through $H^{⊤}\Lambda H+A^{⊤}C^{-1}A$, by setting $Y=\frac{1}{\mu }I_{Q}$, so that A and C satisfy

(24) $$A^{⊤}C^{-1}A=\frac{1}{\mu }I_{Q}-H^{⊤}\Lambda H,$$

where μ>0 is such that $\mu ∥H^{⊤}{\Lambda H∥}_{S}<1$, so that C is positive definite. It follows that the minus logarithm of the conditional distribution of x given z and u is defined up to an additive constant as

(25) $$\left(\forall x\in R^{Q}\right)\left(\forall u\in R^{J}\right)\;J\left(x|u\right)=\frac{1}{2\mu }{∥x∥}^{2}-x^{⊤}\left(H^{⊤}\Lambda z+A^{⊤}C^{-1}u\right)+\Psi \left(Vx\right).$$

Let us make the following change of variables within the Gibbs sampling method:

$$\left(\forall t\in N^{*}\right)\;v^{\left(t\right)}=A^{⊤}C^{-1}u^{\left(t\right)}.$$

According to (15) and (24), we obtain

(26) $$\left(\forall t\in N\right)\;v^{\left(t+1\right)}=\left(\frac{1}{\mu }I_{Q}-H^{⊤}\Lambda H\right)x^{\left(t\right)}+A^{⊤}C^{-1/2}n^{\left(t\right)}\;,$$

where $n^{\left(t\right)}∼N\left(0_{J},I_{J}\right)$. Let us define $\Gamma =\frac{1}{\mu }I_{Q}-H^{⊤}\Lambda H$, which is positive definite. Since $A^{⊤}C^{-1/2}n^{\left(t\right)}$ follows a zero-mean Gaussian distribution with covariance matrix Γ, then

(27) $$\left(\forall t\in N\right)\;v^{\left(t+1\right)}∼N\left(\Gamma x^{\left(t\right)},\Gamma \right)\;,$$

and the new target conditional distribution reads

(28) $$\left(\forall x\in R^{Q}\right)\;J\left(x|v\right)=\frac{1}{2\mu }{∥x-\mu \left(v+H^{⊤}\Lambda z\right)∥}^{2}+\Psi \left(Vx\right).$$

The proposed Gibbs sampling algorithm is then summarized by Algorithm 2.

**Algorithm 2** Gibbs sampler with auxiliary variables in order to eliminate the coupling induced by $H^{⊤}\Lambda H$.**Initialize:**
$x^{\left(0\right)}\in R^{Q}$, $v^{\left(0\right)}\in R^{Q}$, μ>0 such that $\mu ∥H^{⊤}\Lambda H∥<1$1:**for** t=0,1,… **do**2: Generate $v^{\left(t+1\right)}∼N\left(\Gamma x^{\left(t\right)},\Gamma \right)$ where $\Gamma =\frac{1}{\mu }I_{Q}-H^{⊤}\Lambda H$3: Generate $x^{\left(t+1\right)}∼P_{x|v^{\left(t+1\right)},z}$4:**end for**


It can be seen that heterogeneous dependencies initially existing in (11), carried by the likelihood and the prior operators, are now dissociated in the new target distribution (28). Likelihood-related correlations are no longer attached directly to the target signal. They intervene in the conditional law only through the auxiliary variable v and the observation z. In other words, the original problem reduces to solving a denoising problem where the variance of the Gaussian noise is μ. Thereby, the new target distribution (28) is generally easier to sample from compared with the initial one. In particular, one can sample the components independently when the coefficients of the signal are independent in the prior. Otherwise, if Ψ is a smooth function, one can use a Langevin-based MCMC algorithm. For instance, it may be possible to construct an efficient curvature matrix that accounts for the prior correlation and that can be easily manipulated.

Table 1 summarizes the two different cases we have presented here. We would like to emphasize that the approach we propose for adding auxiliary variables according to the structure of the matrix H and Λ is sufficiently generic so that it covers a wide diversity of applications.

It is worth noting that the auxiliary variable could be introduced in the data fidelity term as well as in the prior information. The derivation of the proposed method in (13) allows us to identify classes of models for which our approach can be extended. Obviously, the key requirement is that the term which should be simplified can be written as a quadratic function with respect to some variables. Hence, without completely relaxing the Gaussian requirement, we can extend the proposed method to Gaussian models in which some hidden variables control the mean and/or the variance. This includes, for example, scale mixture of Gaussian models [63,64] such as the alpha-stable family (including the Cauchy distribution), the Bernoulli Gaussian model and the generalized Gaussian distributions, and also Gaussian Markov random fields [55]. In Section 3.2, we will investigate the case of the scale mixture of Gaussian models. When both the likelihood and the prior distribution are Gaussian conditionally to some parameters, the proposed method can be applied to each term as explained in Section 3.3.

Another point to pay attention to is the sampling of the auxiliary variable v. In particular, in Algorithm 2, we should be able to sample from the Gaussian distribution whose covariance matrix is of the form $\left(\frac{1}{\mu }I_{Q}-H^{⊤}\Lambda H\right)$, which is possible for a large class of observation models as discussed in Section 3.4.

### 3.2. Scale Mixture of Gaussian Noise

#### 3.2.1. Problem Formulation

Let us consider the following observation model:

(29) $$\left(\forall i\in \{1,\ldots ,N\}\right)\;z_{i}={\left[Hx\right]}_{i}+w_{i}\;,$$

such that for every i∈{1,…,N},

(30) $$\left\{\begin{array}{cc} w_{i}=0 & \mathrm{if}\;\sigma_{i}=0\\ w_{i}∼N\left(0,\sigma_{i}^{2}\right) & \mathrm{if}\;\sigma_{i}>0 \end{array}\right.\;,$$

where $\left(\sigma_{1},\ldots ,\sigma_{N}\right)$ are independent random variables distributed on $R^{+}$ according to $P_{\sigma }$. Different forms of the mixing distribution $P_{\sigma }$ lead to different noise statistics. In particular, the Cauchy noise is obtained when $\sigma_{1}^{2},\ldots ,\sigma_{N}^{2}$ are random variables following an inverse Gamma distribution. Let $\sigma ={\left[\sigma_{1},\ldots ,\sigma_{N}\right]}^{⊤}$. By assuming that x and σ are independent, the joint posterior distribution of x and σ is given by:

(31) p(x,σ∣z)=p(x∣z)p(σ∣z).

In such a Bayesian estimation context, a Gibbs sampling algorithm is generally adopted to sample alternatively from the distributions $P_{x|\sigma ,z}$ and $P_{\sigma |x,z}$.

In the following, we assume that the set $S_{0}=\left\{\sigma_{1}=\sigma_{2}=\ldots =\sigma_{N}=0\right.\}$ has a zero probability given the vector of observations z. Note that by imposing such rule, we ensure that at each iteration *t* of the Gibbs algorithm, $\sigma^{\left(t\right)}\neq 0_{N}$ almost surely.

Since sampling from $P_{x|\sigma ,z}$ is supposed to be intractable, we propose the addition of auxiliary variables $v\in R^{J}$ that may depend on the variables of interest x and σ according to a given conditional distribution density p(v|x,σ,z)=p(v|x,σ) which satisfies the following conditions:

1. $\int_{R^{J}}p\left(x,\sigma ,v|z\right)dv=p\left(x,\sigma |z\right)$,
2. $\int_{R^{Q}}\int_{R^{N}}p\left(x,\sigma ,v|z\right)\;dxd\sigma =p\left(v|z\right)$,

where p(v|z) should be a valid probability density function.

Using the same arguments as in Section 2.2, these two properties are satisfied provided that p(v|x,σ,z) defines a proper probability density function. It follows that the initial two-step Gibbs iteration is replaced by the following three sampling steps. First, sample $v^{\left(t+1\right)}$ from $P_{v|x^{\left(t\right)},\sigma^{\left(t\right)},z}$ then sample $x^{\left(t+1\right)}$ from $P_{x|\sigma^{\left(t\right)},v^{\left(t+1\right)},z}$, and finally sample $\sigma^{\left(t+1\right)}$ from $P_{\sigma |x^{\left(t+1\right)},v^{\left(t+1\right)},z}$.

#### 3.2.2. Proposed Algorithms

Let D(σ) be the diagonal matrix whose diagonal elements are given by

(32) $$\left(\forall i\in \{1,\ldots ,N\}\right)\;D{\left(\sigma \right)}_{i,i}=\left\{\begin{array}{cc} 0 & \mathrm{if}\;\sigma_{i}=0\\ {\left(\sigma_{i}\right)}^{-2} & \mathrm{if}\;\sigma_{i}>0. \end{array}\right.$$

Note that, since $S_{0}$ has zero probability, we almost surely have

(33) $${∥D\left(\sigma \right)∥}_{S}>0.$$

- Suppose first that there exists a constant ν>0 such that
  (34) $$\left(\forall t⩾0\right)\left(\forall i\in \{1,\ldots ,N\}\right)\;\nu ⩽\sigma_{i}^{\left(t\right)}.$$
  Then, results in Section 3.1 with a Gaussian noise can be extended to scale mixture of Gaussian noise by substituting—at each iteration *t*—$D^{\left(t\right)}$ for Λ, and by choosing $\mu <\nu^{2}$ in Algorithm 1 and ${\mu ∥H∥}_{S}^{2}<\nu^{2}$ in Algorithm 2. The only difference is that an additional step must be added to the Gibbs algorithm to draw samples of the mixing variables $\sigma_{1},\ldots ,\sigma_{N}$ from their conditional distributions given x, v, and z.
- Otherwise, when ν>0 satisfying (34) does not exist, results in Section 3.1 remain also valid when, at each iteration *t*, for a given value of $\sigma^{\left(t\right)}$, we replace Λ by $D(\sigma^{\left(t\right)})$. However, there is a main difference with respect to the case when ν>0, which is that μ depends on the value of the mixing variable $\sigma^{\left(t\right)}$ and hence can take different values along the iterations. Subsequently, μ(σ) will denote the chosen value of μ for a given value of σ. Here again, two strategies can be distinguished for setting ${\left(\mu (\sigma^{\left(t\right)})\right)}_{t\in N}$, depending on the dependencies one wants to eliminate through the DA strategy.

**Alternative I:** Eliminate the Coupling Induced by $D(\sigma^{\left(t\right)})$

A first option is to choose, at each iteration *t*, $\mu (\sigma^{\left(t\right)})$ positive such that

(35) $$\mu \left(\sigma^{\left(t\right)}\right)=\frac{\epsilon }{∥D\left(\sigma^{\left(t\right)}\right){∥}_{S}}=\epsilon {\left(\min{\left(\sigma_{i}^{\left(t\right)}\right)}_{i\in I^{\left(t\right)}}\right)}^{2}\;,$$

with ϵ∈]0,1[ and

(36) $$I^{\left(t\right)}=\left\{i\in \left\{1,\ldots ,N\right\}|\sigma_{i}^{\left(t\right)}>0\right\}.$$

The auxiliary variable is then drawn as follows:

(37) $$v^{\left(t+1\right)}∼N\left(\Gamma \left(\sigma^{\left(t\right)}\right)Hx^{\left(t\right)},\Gamma \left(\sigma^{\left(t\right)}\right)\right)\;,$$

where $\Gamma \left(\sigma^{\left(t\right)}\right)=\frac{1}{\mu (\sigma^{\left(t\right)})}I_{N}-D\left(\sigma^{\left(t\right)}\right)$ is positive definite by construction. The minus logarithm of the posterior density p(x|σ,v,z) is given by

(38) $$\left(\forall x\in R^{Q}\right)\;J\left(x|\sigma ,v\right)=\frac{1}{2\mu (\sigma )}{∥Hx-\mu \left(\sigma \right)\left(v+D(\sigma )z\right)∥}^{2}+\Psi \left(Vx\right).$$

**Alternative II:** Eliminate the Coupling Induced by $H^{⊤}D\left(\sigma^{\left(t\right)}\right)H$

Similarly, in order to eliminate the coupling induced by the full matrix $H^{⊤}D\left(\sigma^{\left(t\right)}\right)H$, $\mu (\sigma^{\left(t\right)})$ can be chosen at each iteration t∈N so as to satisfy

(39) $$\mu \left(\sigma \right)=\frac{\epsilon }{{∥H∥}_{S}^{2}{∥D\left(\sigma \right)∥}_{S}}=\frac{\epsilon }{{∥H∥}_{S}^{2}}{\left(\min{\left(\sigma_{i}^{\left(t\right)}\right)}_{i\in I^{\left(t\right)}}\right)}^{2}\;,$$

with ϵ∈]0,1[ and $I^{\left(t\right)}$ is given by (36). Then, the auxiliary variable is drawn as

(40) $$v^{\left(t+1\right)}∼N\left(\Gamma \left(\sigma^{\left(t\right)}\right)x^{\left(t\right)},\Gamma \left(\sigma^{\left(t\right)}\right)\right),$$

where $\Gamma \left(\sigma^{\left(t\right)}\right)=\frac{1}{\mu (\sigma^{\left(t\right)})}I_{Q}-H^{⊤}D\left(\sigma^{\left(t\right)}\right)H$ is positive definite. The minus logarithm of the posterior density p(x|σ,v,z) then reads

(41) $$\left(\forall x\in R^{Q}\right)\;J\left(x|\sigma ,v\right)=\frac{1}{2\mu (\sigma )}{∥x-\mu \left(\sigma \right)\left(v+H^{⊤}D\left(\sigma \right)z\right)∥}^{2}+\Psi \left(Vx\right).$$

It is worth noting that σ and v are two dependent random variables conditionally to both x and z. The resulting Gibbs samplers, corresponding to Alternatives I and II, respectively, are summarized in Algorithms 3 and 4.

**Algorithm 3** Gibbs sampler with auxiliary variables in order to eliminate the coupling induced by D(σ) in the case of a scale mixture of Gaussian noise.**Initialize:**
$x^{\left(0\right)}\in R^{Q}$, $v^{\left(0\right)}\in R^{N}$, $\sigma^{\left(0\right)}\in R_{+}^{N}$, 0<ϵ<1, $\mu \left(\sigma^{\left(0\right)}\right)=\epsilon {\left(\min{\left(\sigma_{i}^{\left(0\right)}\right)}_{i\in I^{\left(0\right)}}\right)}^{2}$1:**for** t=0,1,… **do**2: Generate $v^{\left(t+1\right)}∼N\left(\Gamma \left(\sigma^{\left(t\right)}\right)Hx^{\left(t\right)},\Gamma \left(\sigma^{\left(t\right)}\right)\right)$ where $\Gamma \left(\sigma^{\left(t\right)}\right)=\frac{1}{\mu (\sigma^{\left(t\right)})}I_{N}-D\left(\sigma^{\left(t\right)}\right)$3: Generate $x^{\left(t+1\right)}∼P_{x|v^{\left(t+1\right)},\sigma^{\left(t\right)},z}$4: Generate $\sigma^{\left(t+1\right)}∼P_{\sigma |x^{\left(t+1\right)},v^{\left(t+1\right)},z}$5: Set $\mu \left(\sigma^{\left(t+1\right)}\right)=\epsilon {\left(\min{\left(\sigma_{i}^{\left(t+1\right)}\right)}_{i\in I^{\left(t+1\right)}}\right)}^{2}$6:**end for**


**Algorithm 4** Gibbs sampler with auxiliary variables in order to eliminate the coupling induced by $H^{⊤}D\left(\sigma \right)H$ in the case of a scale mixture of Gaussian noise.**Initialize:**
$x^{\left(0\right)}\in R^{Q}$, $v^{\left(0\right)}\in R^{Q}$, $\sigma^{\left(0\right)}\in R_{+}^{N}$, 0<ϵ<1, $\mu \left(\sigma^{\left(0\right)}\right)=\epsilon \;{∥H∥}_{S}^{-2}\;{\left(\min{\left(\sigma_{i}^{\left(0\right)}\right)}_{i\in I^{\left(0\right)}}\right)}^{2}$1:**for** t=0,1,… **do**2: Generate $v^{\left(t+1\right)}∼N\left(\Gamma \left(\sigma^{\left(t\right)}\right)x^{\left(t\right)},\Gamma \left(\sigma^{\left(t\right)}\right)\right)$ where $\Gamma \left(\sigma^{\left(t\right)}\right)=\frac{1}{\mu (\sigma^{\left(t\right)})}I_{Q}-H^{⊤}D\left(\sigma^{\left(t\right)}\right)H$3: Generate $x^{\left(t+1\right)}∼P_{x|v^{\left(t+1\right)},\sigma^{\left(t\right)},z}$4: Generate $\sigma^{\left(t+1\right)}∼P_{\sigma |x^{\left(t+1\right)},v^{\left(t+1\right)},z}$5: Set $\mu \left(\sigma^{\left(t+1\right)}\right)=\epsilon {∥H∥}_{S}^{-2}{\left(\min{\left(\sigma_{i}^{\left(t+1\right)}\right)}_{i\in I^{\left(t+1\right)}}\right)}^{2}$6:**end for**


#### 3.2.3. Partially Collapsed Gibbs Sampling

It can be noted that it is generally complicated to sample from $P_{\sigma |x,v,z}$ due to the presence of μ(σ) and D(σ) in the conditional distribution of v. One can replace this step by sampling from $P_{\sigma |x,z}$; that is, directly sampling σ from its marginal posterior distribution with respect to v and conditionally to x and z. In this case, we say that we are partially collapsing v in the Gibbs sampler. One of the main benefits of doing so is that, conditionally to x and z, σ has independent components. However, as σ is sampled independently from v, the constructed Markov chain ${\left(x^{\left(t\right)},\sigma^{\left(t\right)},v^{\left(t\right)}\right)}_{t⩾0}$ may have a transition kernel with an unknown stationary distribution [65]. This problem can also be encountered when the auxiliary variable v depends on other unknown hyperparameters changing along the algorithm, such as prior covariance matrix or regularization parameter when the auxiliary variable is added to the prior instead of the likelihood. However, there are some rules based on marginalization, permutation, and trimming that allow the conditional distributions in the standard Gibbs sampler to be replaced with conditional distributions marginalized according to some variables while ensuring that the target stationary distribution of the Markov chain is maintained. The resulting algorithm is known as the Partially Collapsed Gibbs Sampler (PCGS) [65]. Although this strategy can significantly decrease the complexity of the sampling process, it must be implemented with care to guarantee that the desired stationary distribution is preserved. Applications of PCGS algorithms can be found in [66,67,68].

Assume that, in addition to x, σ, v, we have a vector $\Theta \in R^{P}$ of unknown parameters to be sampled. Note that p(x,σ,Θ,v|z) should be integrable with respect to all the variables. Following [65], we propose the use of a PCGS algorithm that allows us to replace the full conditional distribution $P_{\sigma |x,v,\Theta ,z}$ with its conditional distribution $P_{\sigma |x,\Theta ,z}$ without affecting the convergence of the algorithm to the target stationary law. Algorithm 5 shows the main steps of the proposed sampler. It should be noted that, unlike the standard Gibbs algorithm, permuting the steps of this sampler may result in a Markov chain with an unknown stationary distribution.

**Algorithm 5** PCGS in the case of a scale mixture of Gaussian noise.**Initialize:**
$x^{\left(0\right)}\in R^{Q}$, $v^{\left(0\right)}\in R^{Q}$, $\sigma^{\left(0\right)}\in R_{+}^{N}$, $\Theta^{\left(0\right)}\in R^{P}$1:**for** t=0,1,… **do**2: For all i∈{1,…,N}, generate $\sigma_{i}^{\left(t+1\right)}∼P_{\sigma_{i}|x^{\left(t\right)},\Theta^{\left(t\right)},z}$3: Generate $\Theta^{\left(t+1\right)}∼P_{\Theta |x^{\left(t\right)},\sigma^{\left(t+1\right)},z}$4: Set $\mu (\sigma^{\left(t\right)})$ and $\Gamma (\sigma^{\left(t\right)})$5: Generate $v^{\left(t+1\right)}∼P_{v|x^{\left(t\right)},\sigma^{\left(t+1\right)},\Theta^{\left(t+1\right)},z}$6: Generate $x^{\left(t+1\right)}∼P_{x|v^{\left(t+1\right)},\sigma^{\left(t+1\right)},\Theta^{\left(t+1\right)},z}$7:**end for**


### 3.3. High-Dimensional Gaussian Distribution

The proposed DA approach can also be applied to the problem of drawing random variables from a high-dimensional Gaussian distribution with parameters m and G as defined in (5) and (6). The introduction of auxiliary variables can be especially useful in facilitating the sampling process in a number of problems that we discuss below. In order to make our presentation clearer, an additional index will be added to the variables v and μ, introduced in Section 2.

- If the prior precision matrix $G_{x}$ and the observation matrix H can be diagonalized in the same basis, it can be of interest to add the auxiliary variable $v_{1}$ in the data fidelity term. Following Algorithm 1, let $\mu_{1}>0$ such that $\mu_{1}{∥\Lambda ∥}_{S}<1$ and
  (42) $$v_{1}∼N\left(\left(\frac{1}{\mu_{1}}I_{N}-\Lambda \right)Hx,\frac{1}{\mu_{1}}I_{N}-\Lambda \right).$$
  The resulting conditional distribution of the target signal x given the auxiliary variable $v_{1}$ and the vector of observation z is a Gaussian distribution with the following parameters:
  (43) $$\tilde{G}=\frac{1}{\mu_{1}}H^{⊤}H+G_{x},$$
  (44) $$\tilde{m}={\tilde{G}}^{-1}\left(H^{⊤}\Lambda z+G_{x}m_{x}+H^{⊤}v_{1}\right).$$
  Then, sampling from the target signal can be performed by passing to the transform domain where H and $G_{x}$ are diagonalizable (e.g., Fourier domain when H and $G_{x}$ are circulant).
  Similarly, if it is possible to write $G_{x}=V^{⊤}\Omega V$, such that H and V can be diagonalized in the same basis, we suggest the introduction of an extra auxiliary variable $v_{2}$ independent of $v_{1}$ in the prior term to eliminate the coupling introduced by Ω when passing to the transform domain. Let $\mu_{2}>0$ be such that $\mu_{2}{∥\Omega ∥}_{S}<1$ and let the distribution of $v_{2}$ conditionally to x be given by
  (45) $$v_{2}∼N\left(\left(\frac{1}{\mu_{2}}I_{N}-\Omega \right)Vx,\frac{1}{\mu_{2}}I_{N}-\Omega \right).$$
  The joint distribution of the unknown parameters is given by
  (46) $$p\left(x,v_{1},v_{2}|z\right)=p\left(x|z\right)p\left(v_{1}|x,z\right)p\left(v_{2}|x,z\right).$$
  It follows that the minus logarithm of the conditional distribution of x given z, $v_{1}$, and $v_{2}$ is Gaussian with parameters:
  (47) $$\tilde{G}=\frac{1}{\mu_{1}}H^{⊤}H+\frac{1}{\mu_{2}}V^{⊤}V$$
  and
  (48) $$\tilde{m}={\tilde{G}}^{-1}\left(H^{⊤}\Lambda z+G_{x}m_{x}+H^{⊤}v_{1}+V^{⊤}v_{2}\right).$$
- If $G_{x}$ and H are not diagonalizable in the same basis, the introduction of an auxiliary variable either in the data fidelity term or the prior allows us to eliminate the coupling between these two heterogeneous operators. Let $\mu_{1}>0$ such that $\mu_{1}{∥H^{⊤}\Lambda H∥}_{S}<1$ and
  (49) $$v_{1}∼N\left(\left(\frac{1}{\mu_{1}}I_{Q}-H^{⊤}\Lambda H\right)x,\frac{1}{\mu_{1}}I_{Q}-H^{⊤}\Lambda H\right).$$
  Then, the parameters of the Gaussian posterior distribution of x given $v_{1}$ read:
  (50) $$\tilde{G}=\frac{1}{\mu_{1}}I_{Q}+G_{x}\;,$$
  (51) $$\tilde{m}={\tilde{G}}^{-1}\left(H^{⊤}\Lambda z+G_{x}m_{x}+v_{1}\right).$$
  Note that if $G_{x}$ has some simple structure (e.g,. diagonal, block diagonal, sparse, circulant, etc.), the precision matrix (50) will inherit this simple structure.
  Otherwise, if $G_{x}$ does not present any specific structure, one could apply the proposed DA method to both data fidelity and prior terms. It suffices to introduce an extra auxiliary variable $v_{2}$ in the prior law, additionally to the auxiliary variable $v_{1}$ in (49). Let $\mu_{2}>0$ be such that $\mu_{2}{∥G_{x}∥}_{S}<1$ and
  (52) $$v_{2}∼N\left(\left(\frac{1}{\mu_{2}}I_{Q}-G_{x}\right)x,\frac{1}{\mu_{2}}I_{Q}-G_{x}\right).$$
  Then, the posterior distribution of x given $v_{1}$ and $v_{2}$ is Gaussian with the following parameters:
  (53) $$\tilde{G}=\frac{1}{\mu }I_{Q}$$
  and
  (54) $$\tilde{m}=\mu \left(v_{1}+v_{2}+H^{⊤}\Lambda z+G_{x}m_{x}\right)\;,$$
  where
  (55) $$\mu =\frac{\mu_{1}\mu_{2}}{\mu_{1}+\mu_{2}}.$$

### 3.4. Sampling the Auxiliary Variable

It is clear that the main issue in the implementation of all the proposed Gibbs algorithms arises in the sampling of the auxiliary variable v. The aim of this section is to propose efficient strategies for implementing this step at a limited computational cost, in the context of large-scale problems.

For the sake of generality, we will consider that v follows a multivariate Gaussian distribution with a covariance matrix of the form $\Gamma =\frac{1}{\mu }I_{Q}-H^{⊤}\Lambda H$, where μ>0 satisfies $\mu ∥H^{⊤}{\Lambda H∥}_{S}<1$. Our first suggestion is to set μ such that

(56) $${\mu ∥H∥}_{S}^{2}<\beta <\frac{1}{{∥\Lambda ∥}_{S}},$$

with β>0. For example, one can set $\mu ⩽\frac{\epsilon }{{∥H∥}_{S}^{2}{∥\Lambda ∥}_{S}}$ and $\beta =\frac{\sqrt{\epsilon }}{{∥\Lambda ∥}_{S}}$, where 0<ϵ<1. This allows us to verify the requirement $\mu ∥H^{⊤}{\Lambda H∥}_{S}<1$. Moreover, it leads to

(57) $$\frac{1}{\mu }I_{Q}-H^{⊤}\Lambda H=\frac{1}{\beta }\left(\frac{\beta }{\mu }I_{Q}-H^{⊤}H\right)+H^{⊤}\left(\frac{1}{\beta }I_{N}-\Lambda \right)H.$$

Thus, the sampling step of the auxiliary variable at iteration t∈N can be replaced by the three following steps:

(1) Generate $n^{\left(t+1\right)}∼N\left(0_{N},\frac{1}{\beta }I_{N}-\Lambda \right)$,
(2) Generate $y^{\left(t+1\right)}∼N\left(0_{Q},\frac{1}{\lambda }I_{Q}-H^{⊤}H\right)$ with $\lambda =\frac{\mu }{\beta }⩽\frac{\sqrt{\epsilon }}{{∥H∥}_{S}^{2}}$,
(3) Compute $v^{\left(t+1\right)}=\left(\frac{1}{\mu }I_{Q}-H^{⊤}\Lambda H\right)x^{\left(t+1\right)}+\frac{1}{\sqrt{\beta }}y^{\left(t+1\right)}+H^{⊤}n^{\left(t+1\right)}$,

Hereabove, $y^{\left(t+1\right)}$ and $n^{\left(t+1\right)}$ are independent random variables. One can notice that the sampling problem of the auxiliary variables is now separated into two independent subproblems of sampling from large-scale Gaussian distributions. The first sampling step can usually be performed efficiently. For instance, if Λ is diagonal (e.g., when the model is a scale mixture of Gaussian variables), coefficients $n_{i}^{\left(t+1\right)}$, i∈{1,…,N}, can be drawn separately. Let us now discuss the implementation of the second sampling step, requiring sampling from the zero mean Gaussian distribution with covariance matrix $\frac{1}{\lambda }I_{Q}-H^{⊤}H$.

- In the particular case when H is circulant, sampling can be performed in the Fourier domain. More generally, since $H^{⊤}H$ is symmetric, there exists an orthogonal matrix N such that $NH^{⊤}HN^{⊤}$ is diagonal with positive diagonal entries. It follows that sampling from the Gaussian distribution with covariance matrix $\frac{1}{\lambda }I_{Q}-H^{⊤}H$ can be fulfilled easily within the basis defined by the matrix N.
- Suppose that H satisfies $HH^{⊤}=\nu I_{N}$ with ν>0, which is the case, for example, of tight frame synthesis operators or decimation matrices. Note that $\nu \lambda ⩽\sqrt{\epsilon }<1$. We then have:
  (58) $$\frac{1}{\lambda }I_{Q}-H^{⊤}H={\left(\frac{1}{\sqrt{\lambda }}I_{Q}-\sqrt{\lambda }H^{⊤}H\right)}^{2}+\left(1-\lambda \nu \right)H^{⊤}H.$$
  It follows that a sample from the Gaussian distribution with covariance matrix $\frac{1}{\lambda }I_{Q}-H^{⊤}H$ can be obtained as follows:
  (59) $$y^{\left(t+1\right)}=\left(\frac{1}{\sqrt{\lambda }}I_{Q}-\sqrt{\lambda }H^{⊤}H\right)y_{1}^{\left(t+1\right)}+\sqrt{1-\lambda \nu }H^{⊤}y_{2}^{\left(t+1\right)}\;,$$
  where $y_{1}^{\left(t+1\right)}\in R^{Q}$ and $y_{2}^{\left(t+1\right)}\in R^{N}$ are independent Gaussian random vectors with covariance matrices equal to $I_{Q}$ and $I_{N}$, respectively.
- Suppose that H=MP with $M\in R^{N\times K}$ and $P\in R^{K\times Q}$. Hence, one can set λ>0 and $\tilde{\lambda }>0$ such that
  (60) $${\lambda ∥P∥}^{2}<\tilde{\lambda }<\frac{1}{{∥M∥}^{2}}.$$
  For example, for $\mu =\frac{\epsilon }{{∥P∥}_{S}^{2}{∥M∥}_{S}^{2}{∥\Lambda ∥}_{S}}$, we have $\lambda =\frac{\sqrt{\epsilon }}{{∥P∥}_{S}^{2}{∥M∥}_{S}^{2}}$. Then, we can set $\tilde{\lambda }=\frac{\epsilon^{1/4}}{{∥M∥}_{S}^{2}}$. It follows that
  (61) $$\frac{1}{\lambda }I_{Q}-H^{⊤}H=\frac{1}{\tilde{\lambda }}\left(\frac{\tilde{\lambda }}{\lambda }I_{Q}-P^{⊤}P\right)+P^{⊤}\left(\frac{1}{\tilde{\lambda }}I_{K}-M^{⊤}M\right)P.$$
  It appears that if it is possible to draw merely random vectors $y_{1}^{\left(t+1\right)}$ and $y_{2}^{\left(t+1\right)}$ from the Gaussian distributions with covariance matrices $\frac{\tilde{\lambda }}{\lambda }I_{Q}\;-\;P^{⊤}P$ and $\frac{1}{\tilde{\lambda }}I_{K}-M^{⊤}M$, respectively (for example, when P is a tight frame analysis operator and M is a convolution matrix with periodic boundary condition), a sample from the Gaussian distribution with a covariance matrix $\frac{1}{\lambda }I_{Q}-H^{⊤}H$ can be obtained as follows:
  (62) $$y^{\left(t+1\right)}=\frac{1}{\sqrt{\tilde{\lambda }}}y_{1}^{\left(t+1\right)}+P^{⊤}y_{2}^{\left(t+1\right)}.$$

## 4. Application to Multichannel Image Recovery in the Presence of Gaussian Noise

We now discuss the performance of the proposed DA strategies in the context of restoration of multichannel images (MCIs). Such images are widely used in many application areas, such as medical imaging and remote sensing [69,70,71]. Several medical modalities provide color images, including cervicography, dermoscopy, and gastrointestinal endoscopy [72]. Moreover, in the field of brain exploration with neuro-imaging tools, multichannel magnetic resonance images are widely used for multiple sclerosis lesion segmentation [73]. Indeed, the multicomponent images correspond to different magnetic resonance intensities (e.g., T1, T2, FLAIR). They contain different information on the underlying tissue classes that enable discrimination of the lesions from the background. Multiple channel components typically result from imaging a single scene by sensors operating in different spectral ranges. For instance, about a dozen radiometers may be on-board remote sensing satellites. Most of the time, MCIs are corrupted with noise and blur arising from the acquisition process and transmission steps. Therefore, restoring MCIs is of primary importance as a preliminary step before addressing analysis tasks such as classification, segmentation, or object recognition [74]. Several works dedicated to MCI processing rely on wavelet-based approaches [70,75]. In this section, we propose the adoption of a Bayesian framework for recovering the wavelet coefficients of deteriorated MCI, with the aim of analyzing the performance of the aforementioned hybrid Gibbs samplers.

### 4.1. Problem Formulation

Let us consider the problem of recovering a multicomponent image with *B* components ${\bar{y}}_{1},\ldots ,{\bar{y}}_{B}$ in $R^{R}$ (the images being columnwise reshaped) from some observations $z_{1},\ldots ,z_{B}$ which have been degraded by spatially-invariant blurring operators $B_{1},\ldots ,B_{B}$ and corrupted by independent zero-mean additive white Gaussian noises having the same known variance $\sigma^{2}$. As already stated, here we propose addressing the restoration problem in a transform domain where the target images are assumed to have a sparse representation. Let us introduce a set of tight frame synthesis operators $F_{1}^{*},\ldots ,F_{B}^{*}$ [76] such that

(63) $$\left(\forall b\in \{1,\ldots ,B\}\right)\;{\bar{y}}_{b}=F_{b}^{*}{\bar{x}}_{b}\;,$$

where for every b∈{1,…,B}, $F_{b}^{*}$ is a linear operator from $R^{K}$ to $R^{R}$ with K⩾R and ${\bar{x}}_{b}$ is the vector of frame coefficients of the image ${\bar{y}}_{b}$. Each frame transform operator decomposes the image into *M* oriented subbands at multiple scales with sizes $K_{m}$, m∈{1,…,M}, such that $\sum_{m=1}^{M}K_{m}=K$:

(64) $$\begin{array}{cc} \left(\forall b\in \{1,\ldots ,B\}\right)\;{\bar{x}}_{b}= & ({\bar{x}}_{b,1,1},\ldots ,{\bar{x}}_{b,1,K_{1}},\ldots ,\\ & {\bar{x}}_{b,m,1},\ldots ,{\bar{x}}_{b,m,K_{m}},\ldots ,\\ & {\bar{x}}_{b,M,1},\ldots ,{\bar{x}}_{b,M,K_{M}}{)}^{⊤}. \end{array}$$

Then, the problem can be formulated as (4), that is:

(65) z=Hx+w,

where $w∼N(0_{N},\sigma^{2}I_{N})$, $x={\left[x_{1}^{⊤},\ldots ,x_{B}^{⊤}\right]}^{⊤}\in R^{Q}$, $z={\left[z_{1}^{⊤},\ldots ,z_{B}^{⊤}\right]}^{⊤}\in R^{N}$, $H=BF^{*}\in R^{N\times Q}$ with N=BR, Q=KB,

(66) $$F^{*}=\left(\begin{array}{cccc} F_{1}^{*} & 0 & \ldots & 0\\ 0 & F_{2}^{*} & 0 & 0\\ \ddots & \ddots & \ddots & \ddots \\ 0 & 0 & 0 & F_{B}^{*} \end{array}\right)\;,$$

and

(67) $$B=\left(\begin{array}{cccc} B_{1} & 0 & \ldots & 0\\ 0 & B_{2} & 0 & 0\\ \ddots & \ddots & \ddots & \ddots \\ 0 & 0 & 0 & B_{B} \end{array}\right).$$

We propose exploitation of the cross-component similarities by jointly estimating the frame coefficients at a specific orientation and scale through all the *B* components. In this respect, for every m∈{1,…,M}, for every $k\;\in \;\{1,\ldots ,K_{m}\}$, let $x_{m,k}={\left(x_{b,m,k}\right)}_{1⩽b⩽B}\in R^{B}$ be the vector of frame coefficients for a given wavelet subband *m* at a spatial position *k* through all the *B* components. Note that this vector can be easily obtained through $x_{m,k}=P_{m,k}x$, where $P_{m,k}\in R^{B\times Q}$ is a sparse matrix containing *B* lines of a suitable permutation matrix. To promote the sparsity of the wavelet coefficients and the inter-component dependency, following [70], we assume that for every m∈{1,…,M}, the vectors $x_{m,1}$, *…*, $x_{m,K_{m}}$ are realizations of a random vector following a generalized multivariate exponential power (GMEP) distribution with scale matrix $\Sigma_{m}$, shape parameter $\beta_{m}$, and smoothing parameter $\delta_{m}$. Thus, the minus-log of the prior likelihood is given up to an additive constant by

(68) $$-\log p\left(x|\Sigma_{1},\ldots ,\Sigma_{M}\right)=\sum_{m=1}^{M}\sum_{k=1}^{K_{m}}\psi_{m}(∥\Sigma_{m}^{-1/2}\left(P_{m,k}x-a_{m}\right)∥)\;,$$

where for every m∈{1,…,M}, $a_{m}\in R^{B}$, and for all t∈R, $\psi_{m}\left(t\right)=\frac{1}{2}\;{\left(t^{2}\;+\;\delta_{m}\right)}^{\beta_{m}}$.

Our goal is to compute the posterior mean estimate of the target image as well as the unknown regularization parameters using MCMC sampling algorithms accelerated thanks to our proposed DA strategies. In the following, we will denote by Θ the vector of unknown regularization parameters to be estimated jointly with x in the Gibbs sampling algorithm.

### 4.2. Sampling from the Posterior Distribution of the Wavelet Coefficients

One can expect that the standard sampling algorithms fail to efficiently explore the target posterior not only because of the high dimensionality of the problem, but also because of the anisotropic nature of the wavelet coefficients. In fact, the coefficients belonging to different scales are assumed to follow GMEP priors with different shapes $\beta_{m}$, m∈{1,…,M}. For instance, coefficients belonging to the low-resolution subband are generally assumed to be driven from a Gaussian distribution (i.e., $\beta_{m}=1$), while GMEP priors with very small shape parameter (i.e., $\beta_{m}⩽\frac{1}{2}$) are generally assigned to high-resolution subbands at the first level of decomposition in order to promote sparsity. Therein, one can better explore the directions of interest separately by using different amplitudes than sampling them jointly. However, the observation matrix causes high spatial dependencies between the coefficients, and thus hinders processing the different wavelet subbands independently.

The DA approaches we introduced in Section 3 allow this preconditioning problem to be tackled by adding auxiliary variables to the data fidelity term. More specifically, following Algorithm 2, we propose the introduction of an auxiliary variable $v\in R^{Q}$ such that:

(69) $$v∼N\left(\frac{1}{\sigma^{2}}\left(\frac{1}{\mu }I_{Q}-H^{⊤}H\right)x,\frac{1}{\sigma^{2}}\left(\frac{1}{\mu }I_{Q}-H^{⊤}H\right)\right)\;,$$

where ${\mu ∥B∥}_{S}^{2}{∥F∥}_{S}^{2}<1$.

Since the set of hyperparameters Θ is independent of the auxiliary variable v when conditioned to x, each iteration t∈N of the proposed Gibbs sampling algorithm contains the following steps:

(1)Sample $v^{\left(t+1\right)}$ from $P_{v|x^{\left(t\right)},z}$.(2)Sample $x^{\left(t+1\right)}$ from $P_{x|v^{\left(t+1\right)},\Theta^{\left(t\right)},z}$.(3)Sample $\Theta^{\left(t+1\right)}$ from $P_{\Theta |x^{\left(t+1\right)},z}$.

If B is circulant (by assuming periodic boundary conditions of the blur kernel), the first sampling step can be easily done by passing to the Fourier domain. In particular, if F is orthonormal (that is, $FF^{*}=F^{*}F=I_{Q}$), samples of the auxiliary variables can be obtained by first drawing Gaussian random variables in the Fourier domain and then passing to the wavelet domain. Otherwise, if F is a non-orthonormal transform, sampling can be performed using our results stated in (59) and (62).

Note that in the new augmented space, the restoration problem reduces to a denoising problem with zero-mean Gaussian noise of variance μ, and the posterior density reads:

(70) $$p\left(x|z,v,\Theta \right)\propto \prod_{m=1}^{M}\prod_{k=1}^{K_{m}}\exp\left(-J_{m,k}\left(P_{m,k}x|v\right)\right)\;,$$

where

(71) $$\left(\forall c\in R^{B}\right)\;J_{m,k}\left(c|v\right)=\frac{1}{2\mu \sigma^{2}}∥c-\mu P_{m,k}v-\frac{\mu }{\sigma^{2}}P_{m,k}H^{⊤}{z∥}^{2}+\psi_{m}(∥\Sigma_{m}^{-1/2}\left(c-a_{m}\right)∥).$$

It follows that we can draw samples of vectors $x_{m,k}$, m∈{1,…,M}, $k\;\in \;\{1,\ldots ,K_{m}\}$ in an independent manner. Thus, the resolution of the initial high-dimensional problem of size Q=KB reduces to the resolution of *K* parallel subproblems of size *B*. This is particularly interesting in the case of MCIs where we generally have K≫B.

Instead of processing all the different wavelet coefficients at the same time, the proposed method allows each subproblem to be dealt with independently. This avoids sampling problems related to the heterogeneous prior distribution. Different sampling algorithms may be chosen according to the properties of the target distribution in each subproblem. Specifically, for each sampling subproblem, we propose to use either RW or MALA algorithms [17,77].

In the following, we will discuss the practical implementation of the third step of the Gibbs algorithm; namely, sampling from the posterior distribution of Θ.

### 4.3. Hyperparameters Estimation

#### 4.3.1. Separation Strategy

For every m∈{1,…,M}, $\beta_{m}$ controls the shape of the GMEP distribution, allowing for heavier tails than the Laplace distribution ($\beta_{m}<0.5$) and approaching the normal distribution when $\beta_{m}$ tends to 1. In this work, we assume that for every m∈{1,…,M}, $\beta_{m}$ and $\delta_{m}$ are fixed. Actually, the shape parameter is set to different values with respect to the resolution level, spanning from very small values ($\beta_{m}<0.5$) in order to enforce sparsity in the detail subbands at the first levels of decomposition to relatively higher values ($0.5<\beta_{m}<1$) for detail subband at higher resolution levels, whereas a Gaussian distribution is generally assigned to the low-frequency subband. Furthermore, we set $\delta_{m}$ to a positive small value, ensuring that (78) is differentiable [70]. As already mentioned, the scale matrices ${\left(\Sigma_{m}\right)}_{1\leq m\leq M}$ will be estimated. Let us define $P_{\Sigma_{m}}$ the prior distribution of the scale matrix for each subband m∈{1,…,M} and $p(\Sigma_{m})$ its related density. The associated posterior density reads

(72) $$p\left(\Sigma_{m}|x\right)\propto p\left(\Sigma_{m}\right)\det{\left(\Sigma_{m}\right)}^{-K_{m}/2}\exp\left(-\sum_{k=1}^{K_{m}}\psi_{m}(∥\Sigma_{m}^{-1/2}\left(P_{m,k}x-a_{m}\right)∥)\right).$$

When $\beta_{m}=1$, the GMEP prior reduces to a Gaussian distribution. In this case, a common choice of $P_{\Sigma_{m}}$ is an inverse Wishart distribution and (72) is also an inverse Wishart distribution [78]. However, when $0<\beta_{m}<1$, (72) does not belong to classical families of matrix distributions. In that respect, rather than estimating the scale matrices directly, we resort to a separation strategy. More specifically, we propose the independent estimation of the standard deviations and the correlation terms. Let us decompose the scale matrix for each subband m∈{1,…,M} as follows [79]:

(73) $$\Sigma_{m}=C_{\beta_{m},\delta_{m}}\mathrm{Diag}{\left(s_{m}\right)}^{-1}R_{m}\mathrm{Diag}{\left(s_{m}\right)}^{-1}\;,$$

where $R_{m}\in R^{B\times B}$ is the correlation matrix (whose diagonal elements are equal to 1 and the remaining ones define the correlation between the coefficients and have absolute value smaller than 1), $s_{m}\in R^{B}$ is a vector formed by the square root of the precision parameters (the inverse of standard deviations), and $C_{\beta_{m},\delta_{m}}$ is a multiplicative constant that depends on $\beta_{m}$ and $\delta_{m}$ [70]. The advantage of this factorization can be explained by the fact that the estimation of the correlation matrix will not alter the estimation of the variances. For every m∈{1,…,M}, we decompose the precision vector as follows:

(74) $$s_{m}={\left(C_{\beta_{m},\delta_{m}}\right)}^{1/2}\gamma_{m}^{1/(2\beta_{m})}n_{m}\;,$$

where $\gamma_{m}$ is positive and $n_{m}\in R^{B}$ is a vector of positive coefficients whose sum is equal to 1. Then, $n_{m}$ can be seen as the vector containing positive normalized weights of all the *B* components in the subband *m*.

For simplicity, let us assume that the different components of the image have the same correlation and weights in all subbands; i.e., $R=R_{m}$ and $n_{m}=n$ for every m∈{1,…,M}. Furthermore, let us suppose that n is known. We then have

(75) $$\Theta =\{R,\gamma_{1},\ldots ,\gamma_{M}\}.$$

#### 4.3.2. Prior and Posterior Distribution for the Hyperparameters

One can construct the correlation matrix R by sampling from an inverse Wishart distribution. Specifically, let C∼IW(A,c) where A is an appropriate positive definite matrix of $R^{B\times B}$ and c>0. Then, we can write R=ΔCΔ, where Δ is the diagonal matrix whose elements are given by $\Delta_{i,i}=C_{i,i}^{-1/2}$, for every i∈{1,…,B}. Following [79], we can show that the prior density of R reads:

(76) $$p\left(R\right)\propto det{\left(R\right)}^{-\frac{B+1+c}{2}}\prod_{i=1}^{B}{\left(R^{-1}A\right)}_{i,i}^{-\frac{\nu }{2}}.$$

In the following, we will use the notation R∼SS(A,c) to denote this prior. In particular, when $A=I_{B}$, individual correlations have the marginal density $p\left(\rho_{i,j}\right)={\left(1-\rho_{i,j}^{2}\right)}^{\frac{c-B-1}{2}}$ for every $\left(i,j\right)\in {\left\{1,\ldots ,B\right\}}^{2}$ such that i≠j, which can be seen as a rectangular Beta distribution on the interval [−1,1] with both parameters equal to (c−B+1)/2. For c=B+1, we obtain marginally uniformly distributed correlations, whereas by setting B⩽c<B+1 (or B+1<c), we get marginal priors with heavier (or lighter) tails than the uniform distribution—that is, distributions that promote high correlation values around the extremity of the intervals (or near-zero values), respectively [79]. Thus, the posterior distribution of R is given by

(77) $$p\left(R|x,\gamma_{1},\ldots ,\gamma_{M}\right)\propto \det{\left(R\right)}^{-\frac{B+1+c+Q}{2}}\exp\left(-\Psi (x)\right)\prod_{i=1}^{B}{\left(R^{-1}A\right)}_{i,i}^{-\frac{c}{2}}\;,$$

where

(78) $$\Psi \left(x\right)=\sum_{m=1}^{M}\sum_{k=1}^{K_{m}}\psi_{m}\left(\gamma_{m}^{1/(2\beta_{m})}∥R^{-\frac{1}{2}}\mathrm{Diag}\left(n\right)(P_{m,k}x-a_{m}\right)∥).$$

Here we propose to sample from (77) at each iteration t∈N using an MH algorithm with proposal $\mathrm{SS}(\tilde{A},\tilde{c})$, where $\tilde{A}$ is set to the current value of R at iteration *t* and $\tilde{c}$ is chosen to achieve reasonable acceptance probabilities.

For every m∈{1,…,M}, we assume a Gamma prior for $\gamma_{m}$; that is, $\gamma_{m}\;∼\;G\left(a_{\gamma_{m}},b_{\gamma_{m}}\right)$, where $a_{\gamma_{m}}>0$ and $b_{\gamma_{m}}>0$ [80]. Then, the posterior distribution of $\gamma_{m}$ is given by:

(79) $$p\left(\gamma_{m}|x,R\right)\propto \gamma_{m}^{a_{\gamma_{m}}+\frac{K_{m}}{2\beta_{m}}-1}\exp\left(-b_{\gamma_{m}}\gamma_{m}\right)\exp\left(-\frac{1}{2}\sum_{k=1}^{K_{m}}{\left(\gamma_{m}^{\frac{1}{\beta_{m}}}{∥R^{-\frac{1}{2}}\mathrm{Diag}\left(n\right)\left(P_{m,k}x-a_{m}\right)∥}^{2}+\delta_{m}\right)}^{\beta_{m}}\right).$$

Note that if $\delta_{m}=0$, then (79) reduces to a Gamma distribution with parameters:

(80) $${\tilde{a}}_{\gamma_{m}}=a_{\gamma_{m}}+\frac{K_{m}}{2\beta_{m}},$$

(81) $${\tilde{a}}_{\gamma_{m}}=b_{\gamma_{m}}+\sum_{k}^{K_{m}}{∥R^{-\frac{1}{2}}N\left(P_{m,k}x-a_{m}\right)∥}^{2\beta_{m}}.$$

When $\delta_{m}>0$, sampling from (79) will be performed using an independent MH algorithm with a Gamma proposal of parameters (80) and (81).

#### 4.3.3. Initialization

We propose to set the prior distributions of R, $\gamma_{1},\ldots ,\gamma_{M}$ using empirical estimators from the degraded image. In particular, a rough estimator of R can be computed from the subband containing the low-resolution wavelet coefficients at the highest level of decomposition. In the case when F is orthonormal, the variance of wavelet coefficients of the original image are approximately related to those of the degraded image through:

(82) $$\left(\forall b\in \left\{1,\ldots ,B\right\}\right)\left(\forall m\in \left\{1,\ldots ,M\right\}\right)\;\mathrm{var}\left({\left[F_{b}z_{b}\right]}_{m}\right)=\alpha_{m}\mathrm{var}\left({\left[x_{b}\right]}_{m}\right)+\sigma^{2},$$

where ${\left[.\right]}_{m}$ designates the wavelet coefficients belonging to the subband *m* and $\alpha_{m}$ is a positive constant which depends on the subband index *m* and on the blur matrix. Expression (82) is derived from the considered observation model (65) by assuming a constant approximation of the impulse response of the blur filter in each wavelet subband. Note that $\alpha_{m}$ can be calculated beforehand as follows. Given noise-free data, we compute the original empirical variance for each wavelet subband. Then, we calculate again the new variances of the subbands when the data is blurred using matrix B. The coefficients $\alpha_{m}$ are finally estimated for each wavelet subband by computing the ratio of the two variances by a linear regression. When $\alpha_{m}$ is not too small with respect to 1, estimators of $\mathrm{var}({\left[x_{b}\right]}_{m})$ can be reliably computed from $\alpha_{m}$ and $\mathrm{var}({\left[F_{b}z_{b}\right]}_{m})$ using (82). We propose the use of this method to compute estimators of the variances in subbands at the highest levels of decomposition and then deduce the variances of the remaining subbands by using some properties of multiresolution wavelet decompositions. Note that each detail subband *m* corresponds to a given orientation *l* (horizontal, vertical, diagonal) and a given scale *j* (related resolution level). Actually, the variances of the detail subbands can be assumed to follow a power law with respect to the scale of the subband, which can be expressed as follows [81]:

(83) $$\log\mathrm{var}\left({\left[x_{b}\right]}_{m}\right)=\varrho_{l}j+\varpi_{l}\;,$$

where $\varrho_{l}$ and $\varpi_{l}$ are constants depending on the orientation *l* of the subband *m*. Once the variances of subbands in the two highest levels of decomposition have been computed using (82), we can calculate $\varrho_{l}$ and $\varpi_{l}$ for each orientation *l* using the slope and the intercept of these variances from a log plot with respect to the scale *j*. The remaining variances are then estimated by using (83). We then deduce from these variances an empirical estimator of n, and set the parameters of the prior distributions of $\gamma_{1},\ldots ,\gamma_{M}$.

### 4.4. Experimental Results

In these experiments, we consider the Hydice hyperspectral (https://engineering.purdue.edu/~biehl/MultiSpec/hyperspectral.html) data composed of 191 components in the 0.4 to 2.4μm region of the visible and infrared spectrum. The test image was constructed by taking only a portion of size 256×256 and B=6 components of Hydice using the channels 52, 67, 82, 97, 112, and 127. Hence, the problem dimension was N=393,216. The original image was artificially degraded by a uniform blur of size 5×5 and an additive zero-mean white Gaussian noise with variance $\sigma^{2}=9$ so that the initial signal-to-noise ratio (SNR) was 11.16 dB. We performed an orthonormal wavelet decomposition using the Symlet wavelet of order 3, carried out over three resolution levels, hence M=10 and Q=N. For the subband corresponding to the approximation coefficients (m=10), we chose a Gaussian prior (i.e., $\beta_{m}=2$, $\delta_{m}=0$). For the remaining subbands (m∈{1,…,M−1}), we set $\delta_{m}=10^{-4}$. Moreover, we set $\beta_{m}=0.2$ for the detail subbands corresponding to the lowest level of decomposition, $\beta_{m}=0.4$ for the second level of decomposition, and $\beta_{m}=0.5$ for the third level of decomposition.

**Figure 1:**
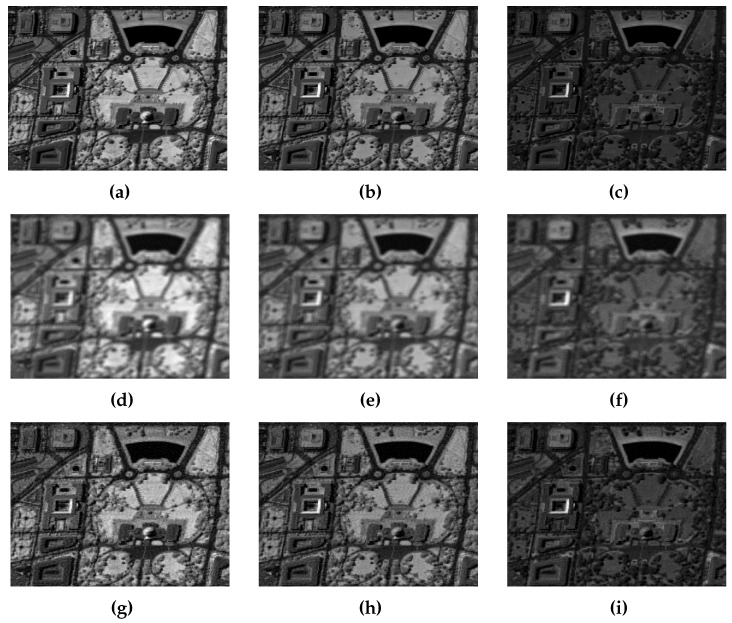
From top to bottom: Original images–Degraded images–Restored images. (**a**) *b* = 2; (**b**) *b* = 4; (**c**) *b* = 6; (**d**) *b* = 2; (**e**) *b* = 4; (**f**) *b* = 6; (**g**) *b* = 2; (**h**) *b* = 4; (**i**) *b* = 6.

We ran the Gibbs sampling Algorithm 2 with a sufficient number of iterations to reach stability. The obtained samples of the wavelet coefficients after the burn-in period were then used to compute the empirical MMSE estimator for the original image. Table 2 reports the results obtained for the different components in terms of SNR, PSNR (peak signal-to-noise ratio), BSNR (blurred signal to noise ratio), and SSIM (structural similarity). It can be noticed that the values of both the objective metrics and the perceptual ones were significantly improved by our method for all the spectral components. For instance, the PSNR values were increased on average by around 4.15 dB, and the SSIM by around 0.23. The achieved gains indicate that the MMSE estimator yielded good numerical results. This can also be corroborated by Figure 1, showing the visual improvements for the different components of the multichannel image. One can observe that all the recovered images were correctly deblurred. Furthermore, small objects were enhanced in all the displayed components.

**Figure 2:**
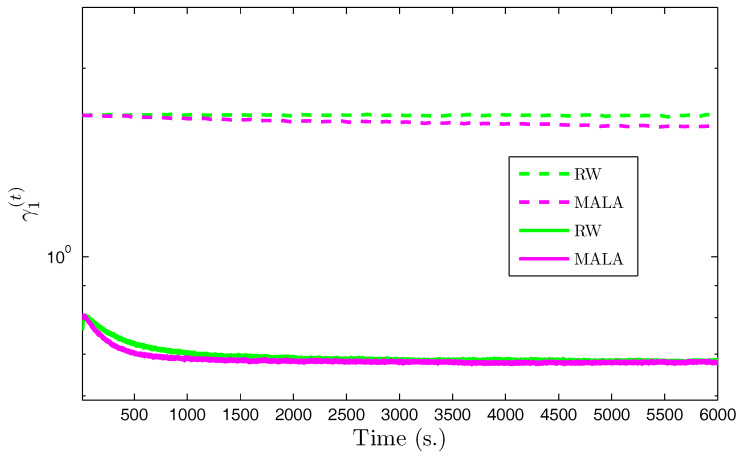
Trace plot of the scale parameter in subband m=1 as time (horizontal subband in the first level of decomposition) with (dashed lines) and without (continuous line) auxiliary variables MALA: Metropolis-adapted Langevin algorithm; RW: random walk.

We propose to compare the performance of the Gibbs sampler with auxiliary variables when the posterior law of the wavelet coefficients is explored using either RW or MALA [17,77] algorithms. We also compared the speed of our proposed approaches with standard RW and MALA without the use of auxiliary variables. Figure 2 shows the evolution—with respect to the computational time—of the scale parameter $\gamma_{m}$ in the horizontal subband for the first level of decomposition using the various algorithms. The results associated with the proposed algorithms appear in solid lines, while those associated with standard algorithms without use of auxiliary variables are in dashed lines. It can be observed that the proposed algorithms reached stability much faster than the standard methods. Indeed, since the problem dimension is large, the stepsize ε in the standard algorithms was constrained to take very small values to allow appropriate acceptance probabilities, whereas in the new augmented space the subproblems dimension was smaller allowing large moves to be accepted with high probability values. Note that the MALA algorithm with auxiliary variables exhibited the best performance in terms of convergence speed. We summarize the obtained samples using the proposed algorithms by showing the marginal means and standard deviations of the hyperparameters in Table 3. It can be noted that the two proposed algorithms provided similar estimation results.

It is worth noting that for larger-dimensional problems (i.e., larger values of *B*), one could further improve the efficiency of the proposed algorithm by exploiting the parallel structure of the sampling tasks.

## 5. Application to Image Recovery in the Presence of Two Mixed Gaussian Noise Terms

### 5.1. Problem Formulation

In this second experiment, we consider the observation problem defined in (29), where H corresponds to a spatially invariant blur with periodic boundary conditions and the noise is a two-term mixed Gaussian variable; i.e., for every i∈{1,…,N}, $w_{i}∼N\left(0,\sigma_{i}^{2}\right)$ such that

(84) $$\sigma_{i}∼\left(1-\beta \right)\delta_{\kappa_{1}}+\beta \delta_{\kappa_{2}}\;,$$

where $\kappa_{1},\kappa_{2}$ are positive, 0<β<1 is the probability that the variance of the noise $\sigma_{i}$ equals $\kappa_{2}$, and $\delta_{\kappa_{1}}$ and $\delta_{\kappa_{2}}$ denote the discrete measures concentrated at the values $\kappa_{1}$ and $\kappa_{2}$, respectively. Model (84) can approximate, for example, mixed impulse Gaussian noise arising in radar, acoustic, and mobile radio applications [82,83]. In this case, the impulse noise is approximated with a Gaussian one with a large variance $\kappa_{2}\gg \kappa_{1}$, and β represents the probability of occurrence of the impulse noise. In the following, we assume without loss of generality that $\kappa_{2}⩾\kappa_{1}$. We address the problem of estimating x, σ, β, $\kappa_{1}$, and $\kappa_{2}$ from the observations z.

### 5.2. Prior Distributions

We propose to use conjugate priors for the unknown variances, namely inverse Gamma distributions; i.e., $\kappa_{i}^{2}∼\mathrm{IG}\left(a_{i},b_{i}\right)$, i∈{1,2}, where $a_{i}$ and $b_{i}$ are positive constants. Here, $a_{1}$, $a_{2}$, $b_{1}$, and $b_{2}$ are set in practice to small values to ensure weakly informative priors. For the occurrence probability β, we chose a uniform prior distribution (i.e., β∼U(0,1)). Furthermore, the target image was assumed to follow a zero-mean Gaussian prior with a covariance matrix $G_{x}^{-1}=\gamma^{-1}{\left(L^{⊤}L\right)}^{-1}$ known up to a precision parameter γ>0; i.e.,

(85) $$p\left(x|\gamma \right)\propto \gamma^{-Q/2}\exp\left(-\frac{\gamma }{2}{∥Lx∥}^{2}\right).$$

**Figure 3:**
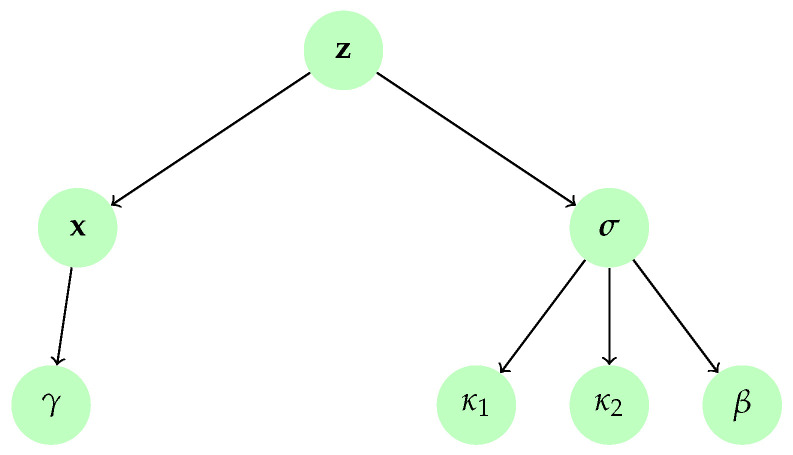
Hierarchical model for image deblurring under two-term mixed Gaussian noise.

Different covariance matrices may be chosen depending on which properties one wants to impose on the estimated image. In this example, we propose to enforce smoothness by setting $L=\delta I_{Q}-\nabla_{2}$, where $\nabla_{2}$ is the circulant convolution matrix associated with a Laplacian filter and δ>0 is a small constant that aims to ensure the positive definiteness of the prior covariance matrix. We further assume that the regularization parameter γ follows an inverse Gamma prior with parameters $a_{\gamma }>0$ and $b_{\gamma }>0$. The resulting hierarchical model is displayed in Figure 3.

#### Posterior Distributions

Given the observation model and the prior distribution, we can deduce that the posterior distribution of the target signal given σ, β, $\kappa_{1}^{2}$, $\kappa_{2}^{2}$, γ, and z is also Gaussian with mean m and precision matrix G given by:

(86) $$G=H^{⊤}DH+\gamma L^{⊤}L,$$

(87) $$m=G^{-1}H^{⊤}Dy,$$

where D is the diagonal matrix with diagonal elements $D_{i,i}=\sigma_{i}^{-2}$, i∈{1,…,N}.

The posterior distributions of the remaining unknown parameters are given by:

- $\left(\forall i\in \{1,\ldots ,N\}\right)\;\sigma_{i}|x,\beta ,\kappa_{1}^{2},\kappa_{2}^{2},z∼\left(1-p_{i}\right)\delta_{\kappa_{1}}+p_{i}\delta_{\kappa_{2}}$ where $p_{i}=\frac{\eta_{i}}{1+\eta_{i}}$ such that
  (88) $$\eta_{i}=\frac{\beta }{1-\beta }\exp\left(-\frac{1}{2}\left(\kappa_{2}^{-2}-\kappa_{1}^{-2}\right){\left({\left[Hx\right]}_{i}-z_{i}\right)}^{2}\right)\frac{\kappa_{1}}{\kappa_{2}},$$
- $\beta |x,z,\sigma ,\kappa_{1}^{2},\kappa_{2}^{2}∼B\left(n_{2}+1,n_{1}+1\right)$, where B is the Beta distribution and $n_{1}$ and $n_{2}$ are the cardinals of the sets $\left\{i\in \left\{1,\ldots ,N\right\},\;|\;\sigma_{i}=\kappa_{1}\right\}$ and $\left\{i\in \left\{1,\ldots ,N\right\},\;|\;\sigma_{i}=\kappa_{2}\right\}$, respectively, so that $n_{1}+n_{2}=N$,
- $\kappa_{1}^{2}|x,\sigma ,\beta ,z∼\mathrm{IG}\left(a_{1}+\frac{n_{1}}{2},b_{1}+\sum_{i|\sigma_{i}=\kappa_{1}}\frac{{\left({\left[Hx\right]}_{i}-z_{i}\right)}^{2}}{2}\right)$,
- $\kappa_{2}^{2}|x,\sigma ,\beta ,z∼\mathrm{IG}\left(a_{2}+\frac{n_{2}}{2},b_{2}+\sum_{i|\sigma_{i}=\kappa_{2}}\frac{{\left({\left[Hx\right]}_{i}-z_{i}\right)}^{2}}{2}\right)$,
- $\gamma |x∼G\left(\frac{Q}{2}+a_{\gamma },\frac{1}{2}{∥Lx∥}^{2}+b_{\gamma }\right)$.

### 5.3. Sampling from the Posterior Distribution of x

In the Gibbs algorithm, we need to draw samples from the multivariate Gaussian distribution of parameters (86) and (87) changing along the sampling iterations. In particular, even if H and L are circulant matrices, sampling cannot be done in the Fourier domain because of the presence of D. In the sequel, we will use the method proposed in Section 3.3 to sample from this multivariate Gaussian distribution. More specifically, we exploit the flexibility of the proposed approach by resorting to two variants. In the first variant, we take advantage of the fact that L and H are diagonalizable in the Fourier domain, and we propose to add the auxiliary variable to the data fidelity term in order to get rid of the coupling caused by D when passing to the Fourier domain. In the second variant, we introduce auxiliary variables for both the data fidelity and the prior terms in order to eliminate the coupling effects induced by all linear operators in the posterior distribution of the target image.

#### 5.3.1. First Variant

We introduce the variable v whose conditional distribution—given the set of main parameters of the problem—is the Gaussian distribution of mean $\left(\frac{1}{\mu }I_{N}-D\right)Hx$ and covariance matrix $\left(\frac{1}{\mu }I_{N}-D\right)$, where $\mu ={\epsilon ∥D∥}_{S}^{-1}$ with 0<ϵ<1. In practice, we set ϵ=0.99. It follows that the new conditional distribution of the target signal is

(89) $$x|\sigma ,\beta ,\kappa_{1}^{2},\kappa_{2}^{2},\gamma ,v,z∼N\left(\tilde{m},{\tilde{G}}^{-1}\right)\;,$$

where $\tilde{m}$ and $\tilde{G}$ are defined as follows:

(90) $$\tilde{G}=\frac{1}{\mu }H^{⊤}H+\gamma L^{⊤}L,$$

(91) $$\tilde{m}={\tilde{G}}^{-1}H^{⊤}\left(H^{⊤}Dz+v\right).$$

It is worth noting that the auxiliary variable v depends on x, and also on σ through μ and D, but does not depend on β, $\kappa_{1}$, $\kappa_{2}$, γ when conditioned to x, σ, and z. Thus, we propose to use the partially collapsed Gibbs sampling algorithm in order to collapse the auxiliary variables in the sampling step of σ. At each iteration t∈N, the proposed algorithm goes through the following steps in an ordered manner:

**AuxV1**(1)Sample ${\left(\kappa_{1}^{2}\right)}^{\left(t+1\right)}$ from $P_{\kappa_{1}^{2}|x^{\left(t\right)},\sigma^{\left(t\right)},\beta^{\left(t\right)},z}$.(2)Sample ${\left(\kappa_{2}^{2}\right)}^{\left(t+1\right)}$ from $P_{\kappa_{2}^{2}|x^{\left(t\right)},\sigma^{\left(t\right)},\beta^{\left(t\right)},z}$.(3)Sample $\beta^{\left(t+1\right)}$ from $P_{\beta |x^{\left(t\right)},\sigma^{\left(t\right)},{\left(\kappa_{1}^{2}\right)}^{\left(t+1\right)},{\left(\kappa_{2}^{2}\right)}^{\left(t+1\right)}}$.(4)Sample $\gamma^{\left(t+1\right)}$ from $P_{\gamma |x^{\left(t\right)}}$.(5)Sample $\sigma^{\left(t+1\right)}$ from $P_{\sigma |x^{\left(t\right)},\beta^{\left(t+1\right)},{\left(\kappa_{1}^{2}\right)}^{\left(t+1\right)},{\left(\kappa_{2}^{2}\right)}^{\left(t+1\right)},z}$.(6)Set $\mu^{\left(t+1\right)}=\epsilon \min{\left(\sigma_{i}^{\left(t+1\right)}\right)}_{1⩽i⩽N}^{-2}$ and sample $v^{\left(t+1\right)}$ from $P_{v|x^{\left(t\right)},\sigma^{\left(t+1\right)}}$.(7)Sample $x^{\left(t+1\right)}$ from $P_{x|\sigma^{\left(t+1\right)},\gamma^{\left(t+1\right)},v^{\left(t+1\right)},z}$.

#### 5.3.2. Second Variant

Another strategy is to introduce two independent auxiliary variables $v_{1}$ and $v_{2}$ in $R^{Q}$ following Gaussian distributions of means $\Gamma_{1}x$ and $\Gamma_{2}x$ and covariance matrices $\Gamma_{1}$ and $\Gamma_{2}$, respectively, where

(92) $$\Gamma_{1}=\frac{1}{\mu_{1}}-H^{⊤}DH$$

and

(93) $$\Gamma_{2}=\frac{1}{\mu_{2}}-L^{⊤}L.$$

In practice, we set $\mu_{1}={\epsilon ∥H∥}_{S}^{-2}{∥D∥}_{S}^{-1}$ and $\mu_{2}=\epsilon {∥L∥}_{S}^{-2}$, where ϵ=0.99. Then, the posterior distribution of x conditioned to σ, β, $\kappa_{1}^{2}$, $\kappa_{2}^{2}$, γ, $v_{1}$, $v_{2}$, and z is Gaussian with mean $\tilde{m}$ and precision matrix $\tilde{G}$ defined as

(94) $$\tilde{G}=\left(\frac{1}{\mu_{1}}+\frac{\gamma }{\mu_{2}}\right)I_{Q}$$

and

(95) $$\tilde{m}=\mu_{1}\mu_{2}{\left(\gamma \mu_{1}+\mu_{2}\right)}^{-1}\left(H^{⊤}Dy+v_{1}+\sqrt{\gamma }v_{2}\right).$$

The auxiliary variable $v_{1}$ depends implicitly on σ through D and μ, but does not depend on the remaining parameters when conditioned to x, σ, and z. Similarly, $v_{2}$ does not depend on σ, β, $\kappa_{1}^{2}$, $\kappa_{2}^{2}$, $v_{1}$, γ when conditioned to x and z. We propose a PCGS algorithm that allows to collapse $v_{1}$ in the sampling step of σ. Each iteration t∈N of the proposed PCGS algorithm is composed of the following arranged sampling steps.

**AuxV2**(1)Sample ${\left(\kappa_{1}^{2}\right)}^{\left(t+1\right)}$ from $P_{\kappa_{1}^{2}|x^{\left(t\right)},\sigma^{\left(t\right)},\beta^{\left(t\right)},z}$.(2)Sample ${\left(\kappa_{2}^{2}\right)}^{\left(t+1\right)}$ from $P_{\kappa_{2}^{2}|x^{\left(t\right)},\sigma^{\left(t\right)},\beta^{\left(t\right)},z}$.(3)Sample $\beta^{\left(t+1\right)}$ from $P_{\beta |x^{\left(t\right)},\sigma^{\left(t\right)},{\left(\kappa_{1}^{2}\right)}^{\left(t+1\right)},{\left(\kappa_{2}^{2}\right)}^{\left(t+1\right)}}$.(4)Sample $\gamma^{\left(t+1\right)}$ from $P_{\gamma |x^{\left(t\right)}}$.(5)Sample $\sigma^{\left(t+1\right)}$ from $P_{\sigma |x^{\left(t\right)},\beta^{\left(t+1\right)},{\left(\kappa_{1}^{2}\right)}^{\left(t+1\right)},{\left(\kappa_{2}^{2}\right)}^{\left(t+1\right)},z}$.(6)Sample $v_{2}^{\left(t+1\right)}$ from $P_{v_{2}|x^{\left(t\right)}}$.(7)Set $\mu_{1}^{\left(t+1\right)}=\epsilon {∥H∥}_{S}^{-2}\min{\left(\sigma_{i}^{\left(t+1\right)}\right)}_{1⩽i⩽N}^{-2}$ and sample $v_{1}^{\left(t+1\right)}$ from $P_{v_{1}|x^{\left(t\right)},\sigma^{\left(t+1\right)}}$.(8)Sample $x^{\left(t+1\right)}$ from $P_{x|\sigma^{\left(t+1\right)},\gamma^{\left(t+1\right)},v_{1}^{\left(t+1\right)},v_{2}^{\left(t+1\right)},z}$.

Note that since H and L are circulant matrices and D is diagonal, sampling the auxiliary variables in the proposed methods can be easily performed following Section 3.4.

### 5.4. Experimental Results

**Figure 4:**
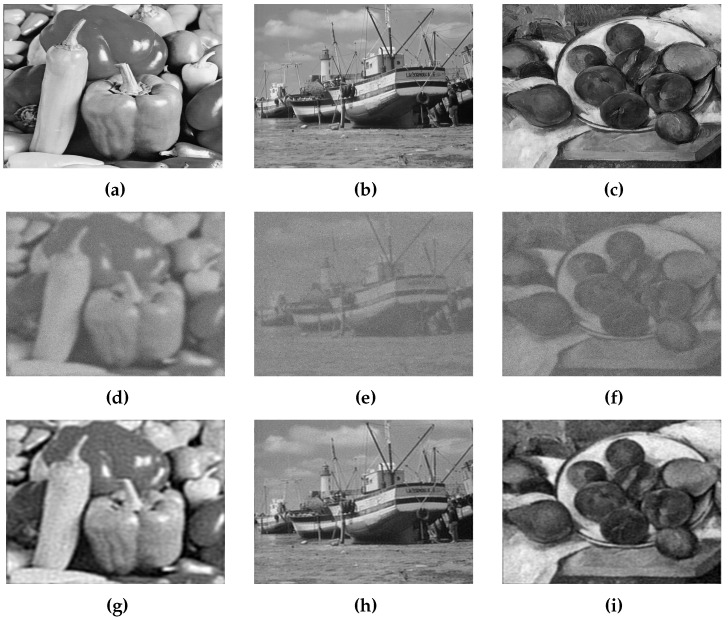
Visual results. From top to bottom: Original images–Degraded images—Restored images. (**a**) ${\bar{x}}_{1}\;\left(512\times 512\right)$; (**b**) ${\bar{x}}_{2}\;\left(512\times 512\right)$; (**c**) ${\bar{x}}_{3}\;\left(512\times 512\right)$; (**d**) $z_{1}$: SNR =13.46 dB, $\kappa_{1}=13$, $\kappa_{2}=40$, β=0.35 h: Gaussian 39×39 std. 4; (**e**) $z_{2}$: SNR = 8.50 dB, $\kappa_{1}=5$, $\kappa_{2}=100$, β=0.25, h: Uniform 5×5; (**f**) $z_{3}$: SNR =7.37 dB, $\kappa_{1}=12$, $\kappa_{2}=70$, β=0.4 h: Gaussian 15×15 std. 1.8; (**g**) ${\hat{x}}_{1}$: SNR =19.35 dB, ${\hat{\kappa }}_{1}=12.98$, ${\hat{\kappa }}_{2}=39.80$ $\hat{\beta }=\;0.35$, $\hat{\gamma }=\;4.8$ × $10^{-3}$; (**h**) ${\hat{x}}_{2}$: SNR =22 dB, ${\hat{\kappa }}_{1}=5.10$, ${\hat{\kappa }}_{2}=100.13$ $\hat{\beta }=\;0.25$, $\hat{\gamma }=1.8$ × $10^{-3}$; (**i**) ${\hat{x}}_{3}$: SNR =18.74 dB, ${\hat{\kappa }}_{1}=12.08$, ${\hat{\kappa }}_{2}=69.89$ $\hat{\beta }=\;0.39$, $\hat{\gamma }=4.7$ × $10^{-3}$.

We consider a set of three test images denoted by ${\bar{x}}_{1}$, ${\bar{x}}_{2}$, and ${\bar{x}}_{3}$, of size 512×512. These images were artificially degraded by a spatially-invariant blur with point spread function *h* and further corrupted with mixed Gaussian noise. The Gibbs algorithms were run for 6000 iterations and a burn-in period of 4000 iterations was considered. Estimators of the unknown parameters were then computed using the empirical mean over the 2000 obtained samples. Visual results are displayed in Figure 4 as well as estimates of hyper-parameters using AuxV1.

**Figure 5:**
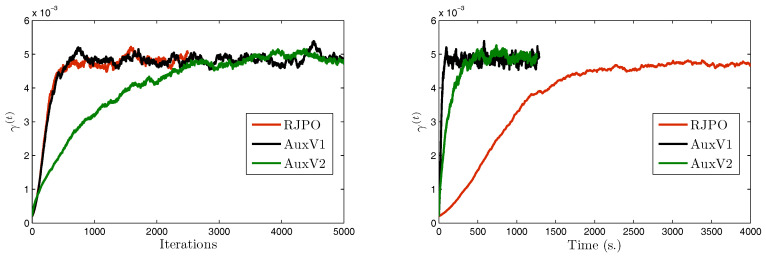
Chains of γ versus iteration/time.

**Figure 6:**
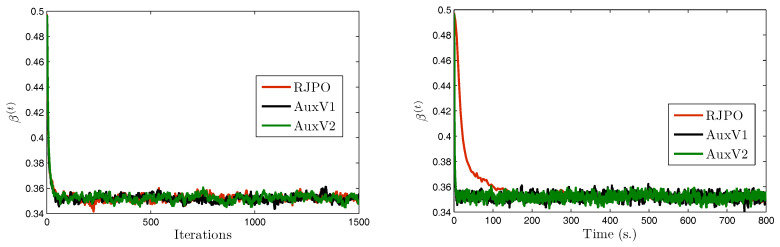
Chains of β versus iteration/time.

**Figure 7:**
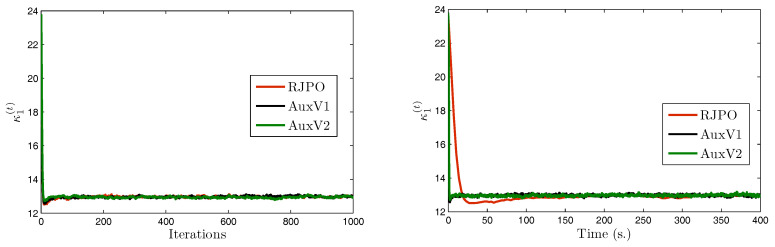
Chains of $\kappa_{1}$ versus iteration/time.

**Figure 8:**
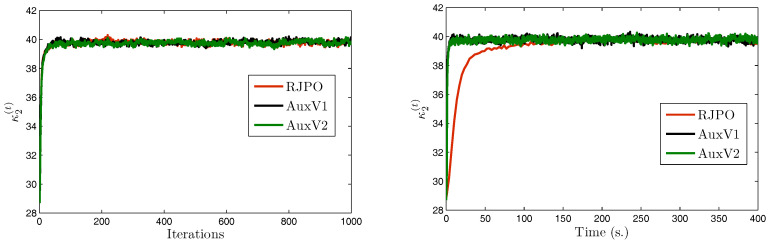
Chains of $\kappa_{2}$ versus iteration/time.

We focus now on image ${\bar{x}}_{1}$ in order to compare the two variants of our proposed method with the Reversible Jump Perturbation Optimization (RJPO) algorithm [32]. For this method, we used the conjugate gradient algorithm as a linear solver at each iteration whose maximal number of iterations and tolerance were adjusted to correspond to an acceptance probability close to 0.9. We used the same initialization for all compared algorithms. Figure 5, Figure 6, Figure 7 and Figure 8 display samples of hyperparameters as a function of iteration or time. By visually examining the trace plots, we can notice that all algorithms were stabilized after an appropriate burn-in period. In particular, RJPO and AuxV1 showed approximately the same iterative behavior, while AuxV2 required about 3000 iterations to reach iconvergence. This corresponds to twice the burn-in length of RJPO and AuxV1. However, each iteration of the RJPO is time consuming since an iterative algorithm is run until convergence at each iteration. Adding auxiliary variables to the model allows the signal to be sampled in a computationally efficient way in the enlarged state space, so that the computational cost of each iteration was highly reduced for both proposed algorithms, and the total time needed to converge was noticeably shortened compared with RJPO. Regarding the stabilization phase, we consider samples generated after the burn-in period (namely, the last 2000 samples for each algorithm). First, we aimed to study the accuracy of estimators of the unknown variables from these samples. More specifically, we computed empirical estimators of the marginal posterior mean and standard deviation of the target parameters as well as those of a randomly chosen pixel $x_{i}$. Table 4 reports the obtained results. It can be noted that parameters β, $\kappa_{1}$, and $\kappa_{2}$ were correctly estimated by all the algorithms, while the remaining parameters had similar estimated values. Second, in order to evaluate the mixing properties of the chains at convergence, we computed an empirical estimation of the mean square jump in stationary state from the obtained samples. This indicator can be seen as an estimation of the average distance between two successive samples in the parameter space. It was computed after the burn-in period $t_{0}=5000$ using P=2000 last samples as follows:

(96) $$MSJ=\sqrt{\frac{1}{P-1}\sum_{t=1}^{P-1}{∥x^{t+t_{0}}-x^{t_{0}+t+1}∥}^{2}}\;.$$

Note that maximizing the mean square jump is equivalent to minimizing a weighted sum of the 1-lag autocorrelations. In Table 5, we show estimates of the mean square jump per second in stationary state, which is defined as the ratio of the mean square jump and the computational time per iteration. This can be seen as an estimation of the average speed of the algorithm for exploring the parameter space at convergence. We also compared the statistical efficiency of the different samplers with respect to RJPO, defined as the mean square jump per second of each sampler over the mean square jump per second of RJPO. We can notice that the speed improvement of the proposed algorithms came at the expense of a deterioration of the quality of the generated samples. In fact, both proposed algorithms yielded lower values of mean square jump than the RJPO algorithm, which indicates that correlation between successive samples was increased. Furthermore, AuxV1 appeared to have better mixing properties compared with AuxV2. However, the generation of every sample in RJPO is very costly, so its efficiency remained globally poorer compared with AuxV1 and AuxV2. The best trade-off between convergence speed and mixing properties of the chain was achieved by the proposed AuxV1 algorithm.

## 6. Conclusions

In this paper, we have proposed an approach for sampling from probability distributions in large-scale problems. By adding some auxiliary variables to the model, we succeeded in separately addressing the different sources of correlations in the target posterior density. We have illustrated the usefulness of the proposed Gibbs sampling algorithms in two application examples. In the first application, we proposed a wavelet-based Bayesian method to restore multichannel images degraded by blur and Gaussian noise. We adopted a multivariate prior model that takes advantage of the cross-component correlation. Moreover, a separation strategy has been applied to construct prior models of the related prior hyperparameters. We then employed the proposed Gibbs algorithm with auxiliary variables to derive optimal estimators for both the image and the unknown hyperparameters. In the new augmented space, the resulting model makes sampling much easier since the coefficients of the target image are no longer updated jointly, but in a parallel manner. Experiments carried out on a set of multispectral satellite images showed the good performance of the proposed approach with respect to standard algorithms. Several issues could be investigated as future work, such as the ability of the proposed algorithm to deal with inter-scale dependencies in addition to the cross-channel ones. In the second application, we have applied the proposed method to the recovery of signals corrupted with mixed Gaussian noise. When compared to a state-of-the-art method for sampling from high dimensional scale Gaussian distributions, the proposed algorithms achieve a good tradeoff between the convergence speed and the mixing properties of the Markov chain, even if the generated samples are not independent. Note that the proposed method can be applied to a wide class of applications in inverse problems—in particular, those including conditional Gaussian models either for the noise or the target signal.

## Figures and Tables

**Table 1:** Different alternatives for adding auxiliary variables.

Problem Source	Proposed Auxiliary Variable	Resulting Conditional Density p(x|z,v)∝exp(−J(x|v))
Λ	$v∼N\left(\left(\frac{1}{\mu }I_{N}-\Lambda \right)Hx,\frac{1}{\mu }I_{N}-\Lambda \right)$	$J\left(x|v\right)=\frac{1}{2\mu }{∥Hx-\mu \left(\Lambda z+v\right)∥}^{2}+\Psi \left(Vx\right)$
$H^{⊤}\Lambda H$	$v∼N\left(\left(\frac{1}{\mu }I_{Q}-H^{⊤}\Lambda H\right)x,\frac{1}{\mu }I_{Q}-H^{⊤}\Lambda H\right)$	$J\left(x|v\right)=\frac{1}{2\mu }{∥x-\mu \left(v+H^{⊤}\Lambda z\right)∥}^{2}+\Psi \left(Vx\right)$

**Table 2:** Restoration results. SNR: signal-to-noise ratio; BSNR: blurred SNR; PSNR: peak SNR; MMSE: minimum mean square error; SSIM: structural similarity.

		b=1	b=2	b=3	b=4	b=5	b=6	**Average**
Initial	BSNR	24.27	30.28	31.73	28.92	26.93	22.97	27.52
	PSNR	25.47	21.18	19.79	22.36	23.01	26.93	23.12
	SNR	11.65	13.23	13.32	13.06	11.81	11.77	12.47
	SSIM	0.6203	0.5697	0.5692	0.5844	0.5558	0.6256	0.5875
MMSE	BSNR	32.04	38.33	39.21	38.33	35.15	34.28	36.22
	PSNR	28.63	25.39	23.98	26.90	27.25	31.47	27.27
	SNR	14.82	17.50	17.60	17.66	16.12	16.38	16.68
	SSIM	0.7756	0.8226	0.8156	0.8367	0.8210	0.8632	0.8225

**Table 3:** Mean and variance estimates of hyperparameters.

		RW	MALA
$\hat{\gamma_{1}}$ ($\gamma_{1}$ = 0.71)	Mean	0.67	0.67
	Std.	(1.63 × $10^{-3}$)	(1.29 × $10^{-3}$)
$\hat{\gamma_{2}}$ ($\gamma_{2}$ = 0.99)	Mean	0.83	0.83
	Std.	(1.92 × $10^{-3}$)	(2.39 × $10^{-3}$)
$\hat{\gamma_{3}}$ ($\gamma_{3}$ = 0.72)	Mean	0.62	0.61
	Std.	(1.33 × $10^{-3}$)	(1.23 × $10^{-3}$)
$\hat{\gamma_{4}}$ ($\gamma_{4}$ = 0.0.24)	Mean	0.24	0.24
	Std.	(1.30 × $10^{-3}$)	(1.39 × $10^{-3}$)
$\hat{\gamma_{5}}$ ($\gamma_{5}$ = 0.40)	Mean	0.37	0.37
	Std.	(2.10 × $10^{-3}$)	(2.42 × $10^{-3}$)
$\hat{\gamma_{6}}$ ($\gamma_{6}$ = 0.22)	Mean	0.21	0.21
	Std.	(1.19 × $10^{-3}$)	(1.25 × $10^{-3}$)
$\hat{\gamma_{7}}$ ($\gamma_{7}$ = 0.0.07)	Mean	0.08	0.08
	Std.	(0.91 × $10^{-3}$)	(1.08 × $10^{-3}$)
$\hat{\gamma_{8}}$ ($\gamma_{8}$ = 0.13)	Mean	0.13	0.13
	Std.	(1.60 × $10^{-3}$)	(1.64 × $10^{-3}$)
$\hat{\gamma_{9}}$ ($\gamma_{9}$ = 0.07)	Mean	0.07	0.07
	Std.	(0.83 × $10^{-3}$)	(1 × $10^{-3}$)
$\hat{\gamma_{10}}$ ($\gamma_{10}$ = 7.44 × $10^{-4}$)	Mean	7.80 × $10^{-4}$	7.87 × $10^{-4}$
	Std.	(1.34 × $10^{-5}$)	(2.12 × $10^{-5}$)
$\det(\hat{R})$ det(R) = 5.79 × $10^{-8}$	Mean	1.89 × $10^{-8}$	2.10 × $10^{-8}$
	Std.	(9.96 × $10^{-10}$)	(2.24 × $10^{-9}$)

**Table 4:** Mean and variance estimates. RJPO: Reversible Jump Perturbation Optimization.

		RJPO	AuxV1	AuxV2
$\hat{\gamma }$ (γ = 5.30 × $10^{-3}$)	Mean	4.78 × $10^{-3}$	4.84 × $10^{-3}$	4.90 × $10^{-3}$
	Std.	(1.39 × $10^{-4}$)	(1.25 × $10^{-4}$)	(9.01 × $10^{-5}$)
$\hat{\kappa_{1}}$ ($\kappa_{1}$ = 13)	Mean	12.97	12.98	12.98
	Std.	(4.49 × $10^{-2}$)	(4.82 × $10^{-2}$)	(4.91 × $10^{-2}$)
$\hat{\kappa_{2}}$ ($\kappa_{1}$ = 40)	Mean	39.78	39.77	39.80
	Std.	(0.13)	(0.14)	(0.13)
$\hat{\beta }$ (β = 0.35)	Mean	0.35	0.35	0.35
	Std.	(2.40 × $10^{-3}$)	(2.71 × $10^{-3}$)	(2.72 × $10^{-3}$)
$\hat{x_{i}}$ ($x_{i}$ = 140)	Mean	143.44	143.19	145.91
	Std.	(10.72)	(11.29)	(9.92)

**Table 5:** Mixing results for the different proposed algorithms. First row: Time per iteration. Second row: Estimates of the mean square jump in stationarity. Third row: Estimates of the mean square jump per second in stationarity. Fourth row: Relative efficiency to RJPO.

	RJPO	AuxV1	AuxV2
*T*(s.)	5.27	0.13	0.12
MSJ	15.41	14.83	4.84
MSJ/*T*	2.92	114.07	40.33
Efficiency	1	39	13.79

## References

[1] Bertero M., Boccacci P. (1998). Introduction to Inverse Problems in Imaging.

[2] Demoment G. (1989). Image reconstruction and restoration: Overview of common estimation structure and problems. IEEE Trans. Acoust. Speech Signal Process..

[3] Marnissi Y., Zheng Y., Chouzenoux E., Pesquet J.C. (2017). A Variational Bayesian Approach for Image Restoration. Application to Image Deblurring with Poisson-Gaussian Noise. IEEE Trans. Comput. Imaging.

[4] Chouzenoux E., Jezierska A., Pesquet J.C., Talbot H. (2015). A Convex Approach for Image Restoration with Exact Poisson-Gaussian Likelihood. SIAM J. Imaging Sci..

[5] Chaari L., Pesquet J.C., Tourneret J.Y., Ciuciu P., Benazza-Benyahia A. (2010). A Hierarchical Bayesian Model for Frame Representation. IEEE Trans. Signal Process..

[6] Pustelnik N., Benazza-Benhayia A., Zheng Y., Pesquet J.C. (1999). Wavelet-Based Image Deconvolution and Reconstruction. Wiley Encyclopedia of Electrical and Electronics Engineering.

[7] Hastings W.K. (1970). Monte Carlo sampling methods using Markov chains and their applications. Biometrika.

[8] Liu J.S. (2001). Monte Carlo Strategies in Scientific Computing.

[9] Gilks W.R., Richardson S., Spiegelhalter D. (1999). Markov Chain Monte Carlo in Practice.

[10] Gamerman D., Lopes H.F. (2006). Markov Chain Monte Carlo: Stochastic Simulation for Bayesian Inference.

[11] Glynn P.W., Iglehart D.L. (1989). Importance sampling for stochastic simulations. Manag. Sci..

[12] Gilks W.R., Wild P. (1992). Adaptive rejection sampling for Gibbs sampling. Appl. Stat..

[13] Neal R.M., Brooks S., Gelman A., Jones G.L., Meng X.L. (2011). MCMC using Hamiltonian dynamics. Handbook of Markov Chain Monte Carlo.

[14] Jarner S.F., Hansen E. (2000). Geometric ergodicity of Metropolis algorithms. Stoch. Process. Appl..

[15] Gilks W.R., Best N., Tan K. (1995). Adaptive rejection Metropolis sampling within Gibbs sampling. Appl. Stat..

[16] Dobigeon N., Moussaoui S., Coulon M., Tourneret J.Y., Hero A.O. (2009). Joint Bayesian Endmember Extraction and Linear Unmixing for Hyperspectral Imagery. IEEE Trans. Signal Process..

[17] Roberts G.O., Gelman A., Gilks W.R. (1997). Weak convergence and optimal scaling or random walk Metropolis algorithms. Ann. Appl. Probab..

[18] Sherlock C., Fearnhead P., Roberts G.O. (2010). The random walk Metropolis: Linking theory and practice through a case study. Stat. Sci..

[19] Roberts G.O., Stramer O. (2002). Langevin diffusions and Metropolis-Hastings algorithms. Methodol. Comput. Appl. Probab..

[20] Martin J., Wilcox C.L., Burstedde C., Ghattas O. (2012). A Stochastic Newton MCMC Method for Large-Scale Statistical Inverse Problems with Application to Seismic Inversion. SIAM J. Sci. Comput..

[21] Zhang Y., Sutton C.A. Quasi-Newton Methods for Markov Chain Monte Carlo. Proceedings of the Neural Information Processing Systems (NIPS 2011).

[22] Girolami M., Calderhead B. (2011). Riemann manifold Langevin and Hamiltonian Monte Carlo methods. J. R. Stat. Soc. Ser. B Stat. Methodol..

[23] Van Dyk D.A., Meng X.L. (2012). The art of data augmentation. J. Comput. Graph. Stat..

[24] Féron O., Orieux F., Giovannelli J.F. (2016). Gradient Scan Gibbs Sampler: An efficient algorithm for high-dimensional Gaussian distributions. IEEE J. Sel. Top. Signal Process..

[25] Rue H. (2001). Fast sampling of Gaussian Markov random fields. J. R. Stat. Soc. Ser. B Stat. Methodol..

[26] Geman D., Yang C. (1995). Nonlinear image recovery with half-quadratic regularization. IEEE Trans. Image Process..

[27] Chellappa R., Chatterjee S. (1985). Classification of textures using Gaussian Markov random fields. IEEE Trans. Acoust. Speech Signal Process..

[28] Rue H., Held L. (2005). Gaussian Markov Random Fields: Theory and Applications.

[29] Bardsley J.M. (2012). MCMC-based image reconstruction with uncertainty quantification. SIAM J. Sci. Comput..

[30] Papandreou G., Yuille A.L. Gaussian sampling by local perturbations. Proceedings of the Neural Information Processing Systems 23 (NIPS 2010).

[31] Orieux F., Féron O., Giovannelli J.F. (2012). Sampling high-dimensional Gaussian distributions for general linear inverse problems. IEEE Signal Process. Lett..

[32] Gilavert C., Moussaoui S., Idier J. (2015). Efficient Gaussian sampling for solving large-scale inverse problems using MCMC. IEEE Trans. Signal Process..

[33] Parker A., Fox C. (2012). Sampling Gaussian distributions in Krylov spaces with conjugate gradients. SIAM J. Sci. Comput..

[34] Lasanen S. (2012). Non-Gaussian statistical inverse problems. Inverse Prob. Imaging.

[35] Bach F., Jenatton R., Mairal J., Obozinski G. (2012). Optimization with sparsity-inducing penalties. Found. Trends Mach. Learn..

[36] Kamilov U., Bostan E., Unser M. Generalized total variation denoising via augmented Lagrangian cycle spinning with Haar wavelets. Proceedings of the IEEE International Conference on Acoustic, Speech and Signal Processing (ICASSP 2012).

[37] Kolehmainen V., Lassas M., Niinimäki K., Siltanen S. (2012). Sparsity-promoting Bayesian inversion. Inverse Prob..

[38] Stuart M.A., Voss J., Wiberg P. (2004). Conditional Path Sampling of SDEs and the Langevin MCMC Method. Commun. Math. Sci..

[39] Marnissi Y., Chouzenoux E., Benazza-Benyahia A., Pesquet J.C., Duval L. Reconstruction de signaux parcimonieux à l’aide d’un algorithme rapide d’échantillonnage stochastique. Proceedings of the GRETSI.

[40] Marnissi Y., Benazza-Benyahia A., Chouzenoux E., Pesquet J.C. Majorize-Minimize adapted Metropolis-Hastings algorithm. Application to multichannel image recovery. Proceedings of the European Signal Processing Conference (EUSIPCO 2014).

[41] Vacar C., Giovannelli J.F., Berthoumieu Y. Langevin and Hessian with Fisher approximation stochastic sampling for parameter estimation of structured covariance. Proceedings of the IEEE International Conference on Acoustic, Speech and Signal Processing (ICASSP 2011).

[42] Schreck A., Fort G., Le Corff S., Moulines E. (2016). A shrinkage-thresholding Metropolis adjusted Langevin algorithm for Bayesian variable selection. IEEE J. Sel. Top. Signal Process..

[43] Pereyra M. (2016). Proximal Markov chain Monte Carlo algorithms. Stat. Comput..

[44] Atchadé Y.F. (2006). An adaptive version for the Metropolis adjusted Langevin algorithm with a truncated drift. Methodol. Comput. Appl. Probab..

[45] Tanner M.A., Wong W.H. (1987). The calculation of posterior distributions by data augmentation. J. Am. Stat. Assoc..

[46] Mira A., Tierney L. On the use of auxiliary variables in Markov chain Monte Carlo sampling. Technical Report, 1997. http://citeseerx.ist.psu.edu/viewdoc/summary?doi=10.1.1.35.7814.

[47] Robert C., Casella G. (2013). Monte Carlo Statistical Methods.

[48] Doucet A., Sénécal S., Matsui T. Space alternating data augmentation: Application to finite mixture of gaussians and speaker recognition. Proceedings of the IEEE International Conference on Acoustic, Speech and Signal Processing (ICASSP 2005).

[49] Févotte C., Cappé O., Cemgil A.T. Efficient Markov chain Monte Carlo inference in composite models with space alternating data augmentation. Proceedings of the IEEE Statistical Signal Processing Workshop (SSP 2011).

[50] Giovannelli J.F. (2008). Unsupervised Bayesian convex deconvolution based on a field with an explicit partition function. IEEE Trans. Image Process..

[51] David H.M. (1997). Auxiliary Variable Methods for Markov Chain Monte Carlo with Applications. J. Am. Stat. Assoc..

[52] Hurn M. (1997). Difficulties in the use of auxiliary variables in Markov chain Monte Carlo methods. Stat. Comput..

[53] Damlen P., Wakefield J., Walker S. (1999). Gibbs sampling for Bayesian non-conjugate and hierarchical models by using auxiliary variables. J. R. Stat. Soc. Ser. B Stat. Methodol..

[54] Duane S., Kennedy A., Pendleton B.J., Roweth D. (1987). Hybrid Monte Carlo. Phys. Lett. B.

[55] Geman S., Geman D. (1993). Stochastic relaxation, Gibbs distributions, and the Bayesian restoration of images. J. Appl. Stat..

[56] Idier J. (2001). Convex Half-Quadratic Criteria and Interacting Auxiliary Variables for Image Restoration. IEEE Trans. Image Process..

[57] Geman D., Reynolds G. (1992). Constrained restoration and the recovery of discontinuities. IEEE Trans. Pattern Anal. Mach. Intell..

[58] Champagnat F., Idier J. (2004). A connection between half-quadratic criteria and EM algorithms. IEEE Signal Process. Lett..

[59] Nikolova M., Ng M.K. (2005). Analysis of half-quadratic minimization methods for signal and image recovery. SIAM J. Sci. Comput..

[60] Bect J., Blanc-Féraud L., Aubert G., Chambolle A. A l1-Unified Variational Framework for Image Restoration. Proceedings of the European Conference on Computer Vision (ECCV 2004).

[61] Cavicchioli R., Chaux C., Blanc-Féraud L., Zanni L. ML estimation of wavelet regularization hyperparameters in inverse problems. Proceedings of the IEEE International Conference on Acoustic, Speech and Signal Processing (ICASSP 2013).

[62] Ciuciu P. (2000). Méthodes Markoviennes en Estimation Spectrale Non Paramétriques. Application en Imagerie Radar Doppler. Ph.D. Thesis.

[63] Andrews D.F., Mallows C.L. (1974). Scale mixtures of normal distributions. J. R. Stat. Soc. Ser. B Methodol..

[64] West M. (1987). On scale mixtures of normal distributions. Biometrika.

[65] Van Dyk D.A., Park T. (2008). Partially collapsed Gibbs samplers: Theory and methods. J. Am. Stat. Assoc..

[66] Park T., van Dyk D.A. (2009). Partially collapsed Gibbs samplers: Illustrations and applications. J. Comput. Graph. Stat..

[67] Costa F., Batatia H., Oberlin T., Tourneret J.Y. A partially collapsed Gibbs sampler with accelerated convergence for EEG source localization. Proceedings of the IEEE Statistical Signal Processing Workshop (SSP 2016).

[68] Kail G., Tourneret J.Y., Hlawatsch F., Dobigeon N. (2012). Blind deconvolution of sparse pulse sequences under a minimum distance constraint: A partially collapsed Gibbs sampler method. IEEE Trans. Signal Process..

[69] Chouzenoux E., Legendre M., Moussaoui S., Idier J. (2012). Fast constrained least squares spectral unmixing using primal-dual interior-point optimization. IEEE J. Sel. Top. Appl. Earth Obs. Remote Sens..

[70] Marnissi Y., Benazza-Benyahia A., Chouzenoux E., Pesquet J.C. Generalized multivariate exponential power prior for wavelet-based multichannel image restoration. Proceedings of the IEEE International Conference on Image Processing (ICIP 2013).

[71] Laruelo A., Chaari L., Tourneret J.Y., Batatia H., Ken S., Rowland B., Ferrand R., Laprie A. (2016). Spatio-spectral regularization to improve magnetic resonance spectroscopic imaging quantification. NMR Biomed..

[72] Celebi M.E., Schaefer G. (2013). Color medical image analysis. Lecture Notes on Computational Vision and Biomechanics.

[73] Criminisi E. (2011). Spatial decision forests for MS lesion segmentation in multi-channel magnetic resonance images. NeuroImage.

[74] Delp E., Mitchell O. (1979). Image compression using block truncation coding. IEEE Trans. Commun..

[75] Khelil-Cherif N., Benazza-Benyahia A. Wavelet-based multivariate approach for multispectral image indexing. Proceedings of the SPIE Conference on Wavelet Applications in Industrial Processing.

[76] Chaux C., Pesquet J.C., Duval L. (2007). Noise Covariance Properties in Dual-Tree Wavelet Decompositions. IEEE Trans. Inf. Theory.

[77] Roberts G.O., Tweedie L.R. (1996). Exponential Convergence of Langevin Distributions and Their Discrete Approximations. Bernoulli.

[78] Murphy K.P. Conjugate Bayesian Analysis of the Gaussian Distribution. Technical Report, 2007. https://www.cs.ubc.ca/~murphyk/Papers/bayesGauss.pdf.

[79] Barnard J., McCulloch R., Meng X.L. (2000). Modeling covariance matrices in terms of standard deviations and correlations, with application to shrinkage. Stat. Sin..

[80] Fink D. (1997). A Compendium of Conjugate Priors. https://www.johndcook.com/CompendiumOfConjugatePriors.pdf.

[81] Flandrin P. (1992). Wavelet analysis and synthesis of fractional Brownian motion. IEEE Trans. Inf. Theory.

[82] Velayudhan D., Paul S. Two-phase approach for recovering images corrupted by Gaussian-plus-impulse noise. Proceedings of the IEEE International Conference on Inventive Computation Technologies (ICICT 2016).

[83] Chang E.S., Hung C.C., Liu W., Yina J. A Denoising algorithm for remote sensing images with impulse noise. Proceedings of the IEEE International Symposium on Geoscience and Remote Sensing (IGARSS 2016).

